# Development of Technology for the Synthesis of Nanocrystalline Cerium Oxide Under Production Conditions with the Best Regenerative Activity and Biocompatibility for Further Creation of Wound-Healing Agents

**DOI:** 10.3390/pharmaceutics16111365

**Published:** 2024-10-25

**Authors:** Ekaterina V. Silina, Victor A. Stupin, Natalia E. Manturova, Elena L. Chuvilina, Akhmedali A. Gasanov, Anna A. Ostrovskaya, Olga I. Andreeva, Natalia Y. Tabachkova, Maxim A. Abakumov, Aleksey A. Nikitin, Alexey A. Kryukov, Svetlana A. Dodonova, Aleksey V. Kochura, Maksim A. Pugachevskii

**Affiliations:** 1 I.M. Sechenov First Moscow State Medical University (Sechenov University), 119991 Moscow, Russia; 2 Pirogov Russian National Research Medical University (RNRMU), 117997 Moscow, Russia; stvictor@bk.ru (V.A.S.); manturovanatali@yandex.ru (N.E.M.); 3 “LANHIT” LLC, 105118 Moscow, Russia; chuvilina.elena@lanhit.ru (E.L.C.); akhmedali@lanhit.ru (A.A.G.); ostrovskaya.a.a@yandex.ru (A.A.O.); a.olga@lanhit.ru (O.I.A.); 4 National University of Science & Technology MISIS, 119049 Moscow, Russia; ntabachkova@gmail.com (N.Y.T.); abakumov1988@gmail.com (M.A.A.); nikitin.chemistry@mail.ru (A.A.N.); 5 Kursk State Medical University, Karl Marx Str., 3, 305041 Kursk, Russia; krukovaa@kursksmu.net (A.A.K.); dodonovasa@kursksmu.net (S.A.D.); 6 Southwest State University, 50 let Oktyabrya Str., 94, 305040 Kursk, Russia; akochura@mail.ru (A.V.K.);

**Keywords:** wound healing, nanotechnology, nanoparticles, cerium dioxide, nanoceria, synthesis methods, industrial conditions, transmission electron microscopy, X-ray diffraction, Raman spectroscopy, mass spectrometry, cells, fibroblasts, keratinocytes, regeneration

## Abstract

**Background/Objectives:** The issue of effective wound healing remains highly relevant. The objective of the study is to develop an optimal method for the synthesis of nanosized cerium oxide powder obtained via the thermal decomposition of cerium carbonate precipitated from aqueous nitrate solution for the technical creation of new drugs in production conditions; the select modification of synthesis under different conditions based on the evaluation of the physicochemical characteristics of the obtained material and its biological activity, and an evaluation of the broad-spectrum effect on cells involved in the regeneration of skin structure as well as antimicrobial properties. **Methods:** Several modes of the industrial synthesis of cerium dioxide nanoparticles (NPs) were carried out. The synthesis stages and the chemical and physical parameters of the obtained NPs were described using transmission electron microscopy (TEM), X-ray diffraction, Raman spectroscopy, and mass spectrometry. The cell cultures of human fibroblasts and keratinocytes were cultured with different concentrations of different nanoceria variations, and the cytotoxicity and the metabolic and proliferative activity were investigated. An MTT test and cell counting were performed. The antimicrobial activity of CeO_2_ variations at a concentration of 0.1–0.0001 M against *Pseudomonas aeruginosa* was studied. **Results:** The purity of the synthesized nanoceria powders in all the batches was >99.99%. According to TEM data, the size of the NPs varied from 1 nm to 70 nm under different conditions and methodologies. The most optimal technology for the synthesis of the nanoceria with the maximum biological effect was selected. A method for obtaining the most bioactive NPs of optimal size (up to 10 nm) was proposed. The repeatability of the results of the proposed method of nanoceria synthesis in terms of particle size was confirmed. It was proven that the more structural defects on the surface of the CeO_2_ crystal lattice, the higher the efficiency of the NPs due to oxygen vacancies. The strain provided the best redox activity and antioxidant properties of the nanoceria, which was demonstrated by better regenerative potential on various cell lines. The beneficial effect of synthesized nanoceria on the proliferative and metabolic activity of the cell lines involved in skin regeneration (human fibroblasts, human keratinocytes) was demonstrated. The antimicrobial effect of synthesized nanoceria on the culture of the most-resistant-to-modern-antibiotics microorganism *Pseudomonas aeruginosa* was confirmed. The optimal concentrations of the nanoceria to achieve the maximum biological effect were determined (10^−3^ M). **Conclusions:** It was possible to develop a method for the industrial synthesis of nanoceria, which can be used to produce drugs and medical devices containing CeO_2_ NPs.

## 1. Introduction

Nanomaterials are a promising area of science and technology development in recent decades. Materials consisting of nanoscale particles of rare-earth metal oxides of the lanthanide group, or including nanoscale components in their composition, are already widely used in industry, because they are able to exhibit new, interesting, and useful properties [1,2,3]. Such features of nanomaterials are explained by the increasing relative proportions of surface atoms in relation to their total number as their particle size decreases. For nanoparticles, almost all atoms are “surface” atoms, so their physical, chemical, and biological activities are high, which is promising for different applications.

Cerium is the most abundant of the rare-earth metals found in the Earth’s crust. Among all rare-earth metals, cerium dioxide (CeO_2_) has attracted much attention in the global nanotechnology market because of its useful applications in catalysts, fuel cells, water purification systems, polishing materials, UV-protective coatings, and the production of specialty ceramics, due to its exceptional thermal and chemical stability, as well as its ease and economy of synthesis [4,5,6,7,8,9,10,11,12,13,14,15]. However, while the advantages of using CeO_2_-based nanomaterials in industry have already become evident, their application in veterinary medicine and general medicine is still in its formative stage. The requirements for nanomaterials with potential biological activity are more stringent than for nanomaterials for industrial applications. These requirements relate, in particular, to their size, structure, and surface morphology. Changes in these parameters when nanoparticles (NPs) enter the biological environment can lead to both a decrease in their biological activity and toxicity to living systems [16,17,18,19,20,21,22].

According to the electronic biomedical library PubMed, the number of publications devoted to the description of synthesis, the analysis of the physicochemical characteristics of NPs, and the analysis of the biological effects of cerium dioxide (nanoceria), is more than 800 in the last 5 years and more than 2500 in the last 20 years. The most promising research from the point of view of creating a biologically effective product (drug) are the results of using cerium oxide NPs to accelerate tissue regeneration simultaneously with antimicrobial activity, which is important for wound healing [22,23,24,25,26,27,28,29,30,31,32,33,34,35,36]. However, despite impressive laboratory results, cerium dioxide NPs have not yet found widespread medical application. One of the reasons for this is the difficulty of the technology of the industrial production of nanoceria, with specified parameters that can provide therapeutic effects when used as part of medical devices.

Nanosized powders of rare-earth metal oxides, including cerium oxide, can be obtained by various methods, but in industrial synthesis they are mainly obtained by the thermal decomposition of rare-earth metal carbonates, hydroxides and oxalates, since this method is the easiest to reproduce, is technologically advanced, is inexpensive, and does not require the use of expensive reagents and equipment, and, importantly for production, allows the obtaining of nanopowders in fairly large batches [7,8,15,37,38,39,40,41,42,43]. It was found that the decomposition of oxalates leads to the formation of larger-sized particles, and hydroxides are poorly filterable, which determines the choice of carbonates as intermediate compounds for subsequent thermal decomposition and obtaining stable CeO_2_ NPs [5,6,7,8,9,10,11,12,13,31,32]. Modern synthesis technologies make it possible to technically create nanosized rare-earth metal oxides in a controlled manner by operating the factors affecting the properties of synthesized materials, such as the concentration of the initial solution, the concentration of the crystallization regulator, the drying and calcination temperatures, and the time of the process [4,7,8,10,12,37,38,39,43,44,45].

Presented today in the scientific literature, other methods of synthesis of lanthanide oxides, including nanoceria (there are hundreds of them, taking into account variants of modifications), unfortunately, in the absolute majority of cases, allow the obtaining of small quantities of substances—grams and micrograms. Even with detailed descriptions in scientific articles, the method of nanoceria synthesis is poorly reproducible and strongly dependent on the “hands” of the experimenter, which is especially noticeable in biomedical research—the obtained biological result does not repeat the result of the authors of the article. This is another important possible reason for the inability of biomedical products based on nanoceria to reach the global market, despite the active research pertaining to their use as a product for biomedicine in the last 20 years.

Thus, the method of the synthesis of the nanopowders of lanthanide oxides, including cerium dioxide, was developed on the basis of the company “LANHIT” LLC, producing nanopowders of rare-earth metals, which allows for the reliable production of the required batches of nanomaterials (tens of kilograms), including those suitable for the further use of synthesized products for biomedical purposes. The synthesis strategy presented at the company and in this study is generally applicable for the inexpensive production of nanoparticles of CeO_2_ with minimal environmental impact.

The aim of this study is to develop an optimal methodology for the synthesis of nanosized cerium oxide powder obtained via the thermal decomposition of cerium carbonate precipitated from a nitrate aqueous solution for the creation of new drugs in production conditions; to choose a modification of the synthesis based on the evaluation of the physicochemical characteristics of the obtained material and its biological activity, as well as to evaluate the effect of different concentrations of nanoceria (from 10^−2^ to 10^−4^ M) on the cells involved in the regenerative process (human fibroblasts, human keratinocytes), as well as their antimicrobial properties.

## 2. Materials and Methods

### 2.1. Synthesis of CeO_2_ Nanoparticles Under Industrial Conditions

The powders of cerium dioxide NPs were synthesized via the thermal decomposition of cerium carbonate obtained by precipitation from nitrate aqueous solution, according to the following reaction equations:

2CeO_2_ + 6HNO_3_ +H_2_O_2_ → 2Ce(NO_3_)_3_ + O_2_↑ + 4H_2_O (1)

2Ce(NO_3_)_3_ + 3(NH_4_)_2_CO_3_ → Ce_2_(CO_3_)_3_ + 6NH_4_NO_3_ (2)

Ce_2_(CO_3_)_3_ → 2CeO_2_ + 3CO_2_ (3)

First, the initial cerium nitrate solution was synthesized using calcined cerium oxide with a purity of 99.99% (LANHIT, Moscow, Russia) and nitric acid of “OSCH 18-4” grade (concentration 70–70.2 wt%, SigmaTech LLC, Moscow, Russia). Then, cerium carbonate was synthesized from purified nitrate, and the concentration of the precipitant (NH_4_)_2_CO_3_ in all the syntheses was 200 g/L (the precipitant was taken in twofold excess). The concentration of the crystallization regulator NH_4_NO_3_ in all the experiments was 375 g/L, which promoted the formation of finer and more homogeneous cerium carbonate particles. The obtained cerium carbonate was filtered, washed with distilled water to negate its nitrate ion content, dried, pulverized, and annealed in a muffle furnace.

For the present study, three modifications of the conditions for the synthesis of nano-cerium oxide were performed (Table 1) with the total volume of the initial solution being 1000 mL. The choice of the temperature regime was based on the known literature data on the thermal decomposition of cerium carbonate (differential thermal analysis). The complete decomposition of the cerium carbonate was observed at 650 °C, but this temperature may promote the high agglomeration of particles. It was found that the minimum calcination temperature of carbonates that guarantees single-phase powder is 450 °C [7,8,10,12,37,38,39,43,44,45]. Since, when the temperature decreases below 400–450 °C, the carbonate does not decompose to cerium oxide (Equation (3)), remaining in the final product as cerium carbonate, the modifications of our synthesis included the burning temperatures of 450 °C and 650 °C for 2 h and 3 h, respectively.

For the synthesis of CeO_2_-I, 200 mL of Ce(NO_3_)_3_ solution with a CeO_2_ concentration of 250 g/L (which, in the resulting solution, amounted to 50 g/L CeO_2_) and 300 mL of crystallization regulator—NH_4_ NO_3_ with a concentration of 375 g/L (112.5 g/L for the total volume)—were placed in the reaction vessel under constant stirring. Then, 500 mL of (NH4)_2_CO_3_ solution with a concentration of 200 g/L (i.e., 100 g/L for the final volume) was uniformly sprayed into the stirred nitrate solution for 10 min. The stirrer speed was 300 rpm. The total volume of the solution was 1 L. Immediately after precipitation, the obtained cerium (III) carbonate precipitate was filtered from the mother liquor on a Buechner funnel (radius of 12 cm) through a double paper filter (blue tape), then washed with double-purified distilled water (volume of about 1.5 L) with a temperature of 40–50 °C to the negative nitrate ion sample. The precipitate was dried at 40 °C to a moisture content of 5–7% (determined by weight method). The dried cerium (III) carbonate was transferred to a quartz cuvette and calcined in a muffle furnace at 650 °C for three hours.

For the synthesis of CeO_2_-II, 200 mL of Ce(NO_3_)_3_ solution with a CeO_2_ concentration of 125 g/L (which amounted to 25 g/L CeO_2_) and 550 mL of crystallization regulator—NH_4_NO_3_ with a concentration of 375 g/L (206.25 g/L for the total volume)—were placed in the reaction vessel under constant stirring. Then, 250 mL of (NH4)_2_CO_3_ solution with a concentration of 200 g/L (i.e., 50 g/L for the final volume) was uniformly sprayed into the stirred nitrate solution for 10 min. The stirrer speed was 300 rpm. The total volume of the solution was 1 L. Immediately after precipitation, the obtained cerium (III) carbonate precipitate was filtered from the mother liquor on a Buechner funnel (radius of 12 cm) through a double paper filter (blue tape), then washed with double-purified distilled water (volume of about 3 L) at a temperature of 20–25 °C to the negative nitrate ion sample. The precipitate was air dried at 20 °C to a moisture content of 5–7%. The dried cerium (III) carbonate was transferred to a quartz cuvette and calcined in a muffle furnace at 450 °C for two hours.

For the synthesis of CeO_2_-III, 200 mL of Ce(NO_3_)_3_ solution with a CeO_2_ concentration of 50 g/L (which amounted to 10 g/L CeO_2_) and 700 mL of crystallization regulator—NH_4_NO_3_ with a concentration of 375 g/L (262.5 g/L for the total volume)—were placed in the reaction vessel under constant stirring. Then, 100 mL of (NH_4_)_2_CO_3_ solution with a concentration of 200 g/L (i.e., 20 g/L for the final volume) was uniformly sprayed into the stirred nitrate solution for 10 min. The stirrer speed was 300 rpm. The total volume of the solution was 1 L. Immediately after precipitation, the obtained cerium (III) carbonate precipitate was filtered from the mother liquor on a Buechner funnel (radius of 12 cm) through a double paper filter (blue tape), then washed with double-purified distilled water (volume of about 2 L) with a temperature of 30–40 °C to the negative nitrate ion sample. The precipitate was dried at 30 °C to a moisture content of 5–7%. During the drying process, the resulting powder was periodically grinded with a fluoroplastic spatula. The dried cerium (III) carbonate was transferred to a quartz cuvette and calcined in a muffle furnace at 450 °C for three hours.

In terms of reproducibility, the synthesis in each of the described groups was performed three times (with initial volumes of 1 L, 1 L, and 10 L) and in all the cases, the physicochemical characteristics of the cerium dioxide NPs powder were not statistically significantly different between the groups.

### 2.2. Study Design

First, the obtained nanocrystalline cerium dioxide powders were investigated using a high-resolution JEOL JEM 2100 transmission electron microscope (JEOL, Japan) with an accelerating voltage of 200 kV (TEM of the first stage). The grain size, structure, and agglomeration of the NPs of all the three studied samples (CeO_2_-I, CeO_2_-II and CeO_2_-III) were evaluated.

The obtained TEM data of the first stage prompted us to create a suspension by dispersing the oxide powder and its subsequent sedimentation into distilled water in order to obtain fine particles and determine the optimal volume of the sol for the subsequent creation of a biomedical product. For this purpose, a 0.1 M suspension (1.72 g of dry nanoceria powder and 100 mL of water) was created and stirred in a magnetic stirrer at 300 rpm for 10 min, followed by sedimentation for 12 h. After 12 h of sedimentation, a precipitate of large agglomerates of nanoceria, occupying 1–2% of the volume, formed at the bottom of the vessel, which we did not use further. We took samples of sols of different fractions (by level) for examination on a transmission electron microscope JEOL JEM-1400 (JEOL, Akishima, Japan) at an accelerating voltage of 120 kV (TEM of the second stage). Three samples from the largest (according to the results of the first stage TEM) NPs of CeO_2_-I powder were examined: at the level of the upper fraction (top 10%), at the level of the lower fraction (at 80–90%, i.e., 10–20% above the precipitation zone), and in the middle (in the middle between the lower and upper fractions).

After performing the TEM of the second step, the nanoceria sols after the sedimentation stage were dried to obtain dry powders, which were examined by X-ray diffraction analysis using a Rigaku UltimaI VcCoKα X-ray diffractometer with the emission at the diffraction angles Θ ranging from 10 to 120° at a scanning speed of 0.1° in 4 s. The nanoceria sols were dried under a vacuum (20 mbar) using a rotary evaporator to obtain dry powders.

Also, the primary nanoceria powders were characterized via Raman spectroscopy using an OMEGA Scope^TM^ Raman spectrometer (AIST-NT Inc., Edison, NJ, USA) with 600 line/mm diffraction grating.

The concentration of CeO_2_ at the level of the different fractions was determined by gravimetry using a muffle furnace (900 °C).

The physicochemical studies were completed by mass spectrometry performed on a JMS-01-BM2 mass spectrometer (JEOL, Akishima, Japan) to determine the purity of the obtained product.

For further biomedical studies on cell lines (human fibroblasts, human keratinocytes), the upper part (up to 45%) of the nano-sols fraction was used. Sols were prepared at concentrations of 10^−2^ M, 10^−3^ M, and 10^−4^ M, according to CeO_2_. Thus, the study of 10 groups was performed: CeO_2_-I at concentrations of 10^−2^ M, 10^−3^ M, and 10^−4^ M; CeO_2_-II at concentrations of 10^−2^ M, 10^−3^ M, and 10^−4^ M; CeO_2_-III at concentrations of 10^−2^ M, 10^−3^ M, and 10^−4^ M; and a control group of distilled water, with which dilutions were performed. In addition, an additional study of the 72 h co-cultivation of CeO_2_-I powder at concentrations of 10^−2^ M, 10^−3^ M, and 10^−4^ M, but without the sedimentation step (i.e., a suspension containing cerium dioxide powder obtained immediately after precipitation without sedimentation, therefore containing all three fractions) was performed.

Sols containing 0.1 M, 0.01 M, 0.001 M, and 0.0001 M CeO_2_ for each of the three synthesis modifications were prepared for microbiological studies.

### 2.3. Physicochemical Methods of Research

#### 2.3.1. Transmission Electron Microscopy

The structure of the powders was studied via transmission electron microscopy using two microscopes—JEM 2100 at an accelerating voltage of 200 kV (nanoceria powders) and JEOL JEM-1400 at an accelerating voltage of 120 kV (sols). TEM studies were performed at the National University of Science and Technology «MISIS».

Standard copper grids covered with amorphous carbon were used for the preparation of powders for TEM.

The JEOL JEM 2100 transmission electron microscope (JEOL, Akishima, Japan) had a maximum accelerating voltage of 200 kV, with a direct magnification of up to 1.5 million times. A LaB_6_ cathode was used as an electron source. The minimum diameter of the electron beam in the lumen mode was 20 nm, which made it possible to obtain a diffraction pattern from an area of the same diameter in the microbeam mode. For high-resolution imaging, the samples were oriented relative to the primary beam with an accuracy of fractions of a degree. For this purpose, a sample holder was used with the goniometer stage tilted along two axes (*x*-axis ± 60°, and *y*-axis ± 25°). The accelerating voltage stability was 2 × 10^−6^ min^−1^. The stability of the objective lens current was 1 × 10^−6^ min^−1^.

Overview images were obtained at low magnifications (×5000–20,000). For a more detailed study of the structure and the observation of the traces of the atomic planes, direct magnification ×400,000–600,000 was used; the maximum approximate ruler on the microphotographs was 5 nm. Selector apertures were used to select areas for obtaining diffraction patterns.

During the TEM of the second stage, sols of the upper, middle and lower fractions were studied in native form by applying drops to a slide with an amorphous carbon substrate. Distilled water was added to the sample of the lower fraction and the resulting suspension was treated with an ultrasound for 2 min. After that, 10 µL of the obtained colloidal solution was applied to the surface of the grid and we waited for its complete drying at room temperature. Light-field and dark-field images of the nanoceria sols were obtained on a JEOL JEM-1400 transmission electron microscope (JEOL, Akishima, Japan) at an accelerating voltage of 120 kV. For this purpose, 10 μL of aqueous colloid of particles was applied to the surface of a copper grid coated with formvar and we waited for the complete drying of the solvent, after which the grid was placed in the microscope column and particle morphology studies were performed.

The average particle size was estimated manually using ImageJ2 and Origin2021 software. For this purpose, the size intervals were selected based on the obtained particle size values (at least 200). Then, the particles of a particular size were attributed to their corresponding interval, after which a histogram of the dependence of the particle size on their total number was plotted. For the obtained histogram, the best NPs’ size distribution functions were determined, from which the values of the average size and standard deviation were taken.

#### 2.3.2. Other Physicochemical Methods

X-ray diffraction images of the powders were obtained at room temperature using a Rigaku Ultima IV CoKα X-ray diffractometer at diffraction angles Θ from 10 to 120° at a scan rate of 0.1° in 4 s. The average coherent scattering region (D), corresponding to the crystallite size, was calculated using the Scherrer formula:

*D* = 0.94 λ/*βcos*,

where β is the full width at half maximum and θ is the Bragg angle.

Raman spectra were measured at room temperature using an Omega Scope TM Raman spectrometer (AIST-NT Inc., Edison, NJ, USA) with a 600 line/mm diffraction grating. Excitation was performed by a laser diode with a wavelength of 473 nm (excitation energy of 2.62 eV); the power was 3.6 mW and the size of the focused light spot on the sample surface on the sample surface was about 500 nm. The spectral resolution was 3.1 cm^−1^. The frequency of the recorded radiation on the lower energy side was limited to 150 cm^−1^. Although the measurement range of Raman spectroscopy was from 150 cm^−1^ to 5000 cm^−1^, significant peaks from the samples were only identified within the range of 350 cm^−1^ to 1600 cm^−1^.

The concentration of CeO_2_ in different fractions was determined by gravimetry using a muffle furnace (900 °C). 

The test on total impurities evaluation was made by Spark Source Mass Spectrometry. The JMS-01-BM2 double focusing mass spectrometer manufactured by JEOL (Japan) was applied. The high-resolution mass spectra were photographed on Ilfrod-Q plates. A Joyce Loebl (United Kingdom) MDM6 microdensitometer and a NOVA 4 (USA) on-line mini-computer were used for the mass spectrum lines identification. Quantity estimation was calculated by the original GC–MS Lab software. The relative standard deviation was 0.15–0.30. The noble gasses and transuranium elements are not tabulated in the table, because their concentrations are lower than the 0.01 ppm detection limits. The impurities’ contents are presented as parts per million weight to the metal matrix (1 ppm = 0.0001%).

### 2.4. Biological Methods of Research on Cell Lines

Two cell lines were used—human fibroblasts (BJTERT line) and human keratinocytes (HaCaT line). These cells were chosen for the study because they are essential participants in the physiological healing of skin wounds. We chose human fibroblasts and human keratinocytes because our main goal in our work is to develop drugs for humans.

The studies were performed after 72 h of co-cultivation with 10 vol% of nanoceria sols. The effects on the proliferative activity and potential cytotoxicity were investigated by assessing the change in metabolic activity in the MTT test and via direct cell counting with the percentage of the dead cells in the cell counter. We applied these different complementary methods to determine the most effective variant and concentration of nanoceria with the best stimulation of metabolic activity (MTT test) and proliferation (cell counter) in the absence of cell death.

#### 2.4.1. Cell Lines and Cell Culture

Human fibroblasts of the BJTERT cell line, which were a modified primary human fibroblast line obtained by introducing a specific genetic element, were the human catalytic subunit of the telomerase enzyme (telomerase reverse transcriptase—hTERT). The source of the cells was the foreskin of newborn boys. The lineage origin of the cells was the American Type Culture Collection (ATCC) (Manassas, VA, USA).

Human keratinocytes (HaCaT cell line), which are spontaneously immortalized non-carcinogenic human keratinocytes capable of unrestricted division but exhibiting normal differentiation, were used [46,47]. The cell source was adult human skin. The lineage origins were the Federal State Budgetary Institution, the Medical Research Center of Oncology, named after the N.N. Blokhin National Medical Research Center for Oncology, and the Ministry of Health of the Russian Federation (Moscow, Russia).

The cells were cultured in culture Petri dishes (SPL Life Sciences, Pocheon, Republic of Korea) in a DMEM medium with high glucose content (at least 4.5 g/L) (PanEco, Moscow, Russia), supplemented with 10% fetal calf serum (Global Kang Biotechnology, Building, China), 146 mg glutamine (PanEco, Moscow, Russia) and 1% penicillin, and 1% streptomycin (PanEco, Moscow, Russia). The cell cultures were incubated in a CO_2_ incubator (Binder, Baddeckenstedt, Germany) under standard controlled conditions (5% CO_2_, 37 °C) and controlled humidity. The passage of BJTERT cells was performed every 7 days when the fibroblasts reached 100% confluency according to the standard protocol; the medium was changed every 3 days.

The passage of keratinocytes (HaCaT line) was performed every 3 days according to the standard protocol. For the experiment, the cells were seeded in 24-well plates (SPL Life Sciences, Pocheon, Korea) using a DMEM medium according to the standard protocol in suspensions of 5.0 × 10^4^/mL for the BJ TERT line and 7.0 × 10^4^/mL for the HaCaT line, respectively. After 24 h, the test substances (in the nanocrystalline cerium oxide of different synthesis methods and in different concentrations according to the experiment design) were added and co-incubation was continued for the next 72 h. The initial solvent, distilled water, was added as a control.

#### 2.4.2. MTT Test Methodology

An MTT test was performed according to the previously described method [23,36]. Briefly, after the incubation time, the culture medium was removed and 3% MTT reagent (PanEco, Russia) prepared from 5 mg/mL stock solution was added and incubated under thermostat conditions at a maintained environment temperature of 37 °C. Next, the MTT working solution was drained and dimethyl sulfoxide (Pan Reac Appli Chem, Darmstadt, Germany) was added; the plate was incubated on an Elmi-S4 rocking platform (Latvia) at room temperature for 5 min until the dissolution of formazan and the acquisition of the homogeneity of the solution in the well. After that the optical density was measured on a spectrophotometer Multiscan Labsystems (Vantaa, Finland) at a wavelength of 540 nm. The final result was expressed in relative optical density (OD) units.

Each sample (each nanoceria group) was examined in 10 wells. Injection water was used as a control, which was added in a similar amount (100 µL to a total volume of 1000 µL), also in 10 wells.

#### 2.4.3. Quantitative Cell Counting and Assessment of Cytotoxicity

Quantitative cell counting with cytotoxicity determination based on the assessment of the cell membrane’s permeability using trypan blue vitreal dye was performed according to the previously described method [36]. Cell counting and the measurement of the live/dead ratio were performed automatically using a Countess II Automated Cell Counter (Thermo Scientific, Waltham, MA, USA). Briefly, the culture medium in which the cells grew for 108 h of incubation was withdrawn from the well, then the well was washed with phosphate buffer, after which the buffer was also withdrawn for evaluation. Next, depending on the cell line, cell detachment was performed according to the following protocol. Versen/trypsin solution (PanEco, Moscow, Russia) was added to the BJTERT fibroblasts in a ratio of 4:1 and incubated under thermostatic conditions for 1 min, then the detached cells were taken into the previously selected samples from the corresponding wells. The detachment of the keratinocytes was performed in two stages—for initial soft detachment, Versen solution was added and incubated at 37 °C for a minute, then for complete detachment, Versen/trypsin solution was added to the well in the ratio 1:1 and also incubated at 37 °C for another 4 min. Similarly to the actions with fibroblasts, detached keratinocytes were taken into the previously selected samples from the well. The samples were carefully pipetted, then 0.4% trypan blue solution was added and direct cell counting was performed using appropriate slides. As a result, the total concentration of the cells in a unit volume was calculated by expressing the total in number of cells (×10^4^ cells), and the percentage of live and dead cells was calculated.

### 2.5. Microbiological Research

To determine the antimicrobial activity, the test strain *Pseudomonas aeruginosa* (ATCC 9027) from the Federal Budgetary Institution of Science’s “State Scientific Center of Applied Microbiology and Biotechnology”’s state collection of pathogenic microorganisms and cell cultures, “SCPM—Obolensk”, was used (Obolensk, Russia).

The antimicrobial activity was determined by the method of diffusion into agar on a dense nutrient medium by analyzing the growth inhibition of the test microorganisms used to determine the antimicrobial effect of the drugs. Cultures of the test microorganism strains were grown on a dense medium (meat peptone agar) at 37 °C ± 2 °C for 20 h.

A microbial load of 500,000 microorganisms in 1 mL was chosen for the experiment to determine antimicrobial activity. For this purpose, 0.2 mL of 1,000,000,000 microorganism suspension (which corresponds to 10 units according to the McFarland standard) was added to 400 mL of meat–peptone agar that was heated to 50 °C, and 25 mL was poured into sterile Petri dishes.

Petri dishes with solidified seeded medium were thermostated to remove condensation, after which 7 mm diameter wells were cut. Each well was filled with 0.1 mL of the test sample. To reduce the influence of time fluctuations between the application of the test substance solution, the dishes were incubated at room temperature for 1 h, then incubated at 36 °C for 18 h. At the end of this period, the growth retardation zones of the test microbes were measured.

### 2.6. Statistical Processing

Statistical processing was carried out using a SPSS 25.0 statistical program (IBM Corp., Armonk, NY, USA). First of all, the normality of the distributions of the MTT test parameters and the cell counts for each of the samples was assessed using the Kolmogorov–Smirnov and Shapiro–Wilk criteria. The normality test confirmed obedience to the law of normal distribution (*p* > 0.05). The descriptive statistics of the continuous quantitative measures were presented as the mean, standard deviation, standard error, deviation, mean error, 95% confidence interval for the mean (95 CI), minimum, and maximum.

A one-factor ANOVA analysis of variance was performed for the comparative analysis of the different subgroups. Posterior multiple comparisons were performed using Dunnett’s test (for comparison with the controls), as were Bonferroni’s test and Duncan’s test, so that the samples could be categorized into homogeneous subgroups, with the most promising cells in terms of stimulation being selected.

Differences were considered statistically significant when the *p*-value < 0.05.

## 3. Results

### 3.1. Results of Transmission Electron Microscopy

#### 3.1.1. TEM of Initial Powders of Cerium Dioxide Nanoparticles

The grain size, structure, and agglomeration of the NPs of all three of the studied samples (CeO_2_-I, CeO_2_-II and CeO_2_-III) were investigated using a high-resolution JEOL JEM 2100 transmission electron microscope (JEOL, Japan) with an accelerating voltage of 200 kV, and the grain size, structure, and agglomeration of the NPs were evaluated.

**Figure 1:**
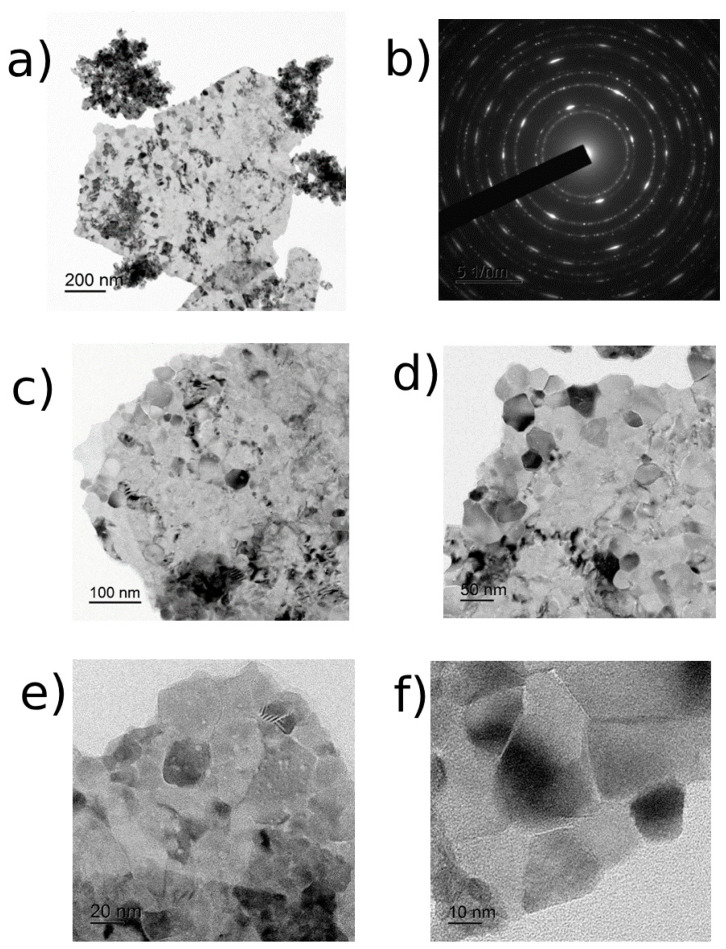
TEM images of CeO_2_-I powder: (**a**) overview image of agglomerate and (**b**) its electronogram; (**c**–**f**) enlarged image before visualization of individual nanoparticles with scale bar from 100 nm to 10 nm.

**Figure 2:**
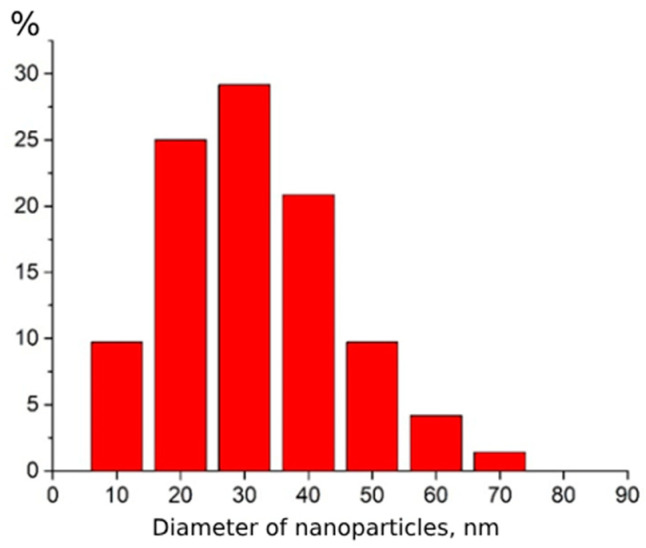
Grain size distribution of CeO_2_-I powder nanoparticles.

The study of CeO_2_-I powder (Figure 1) showed that it represented lamellar particles consisting of polycrystalline nanosized grains. The arrangement of the reflexes on the electronogram obtained from the individual agglomerates corresponded to the CeO_2_ phase. The plate sizes varied widely from 100 nm to micron units. Figure 1a,b shows an image of an individual plate (agglomerate), an electronogram, and a high-resolution image of polycrystalline grains. The size distribution of the NPs is shown in Figure 2. The grain size of the particles ranged from 10 to 70 nm. The maximum of the grain size distribution corresponded to 30 nm.

**Figure 3:**
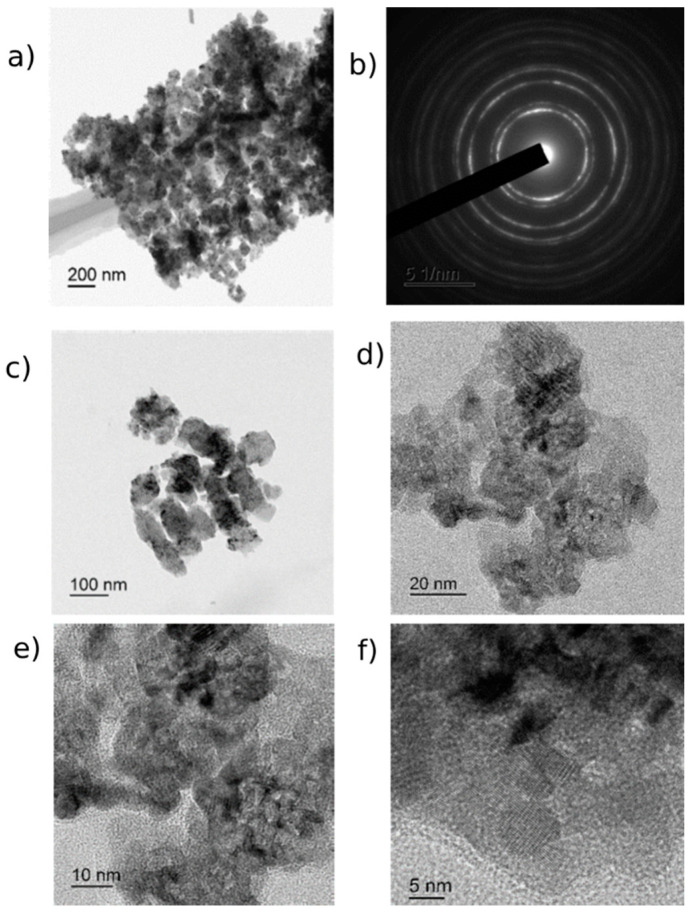
TEM image of CeO_2_-II powder particles: (**a**) overview image of agglomerate and (**b**) electronogram from this agglomerate; (**c**–**f**) enlarged image of individual nanoparticles with scale bar from 100 nm to 5 nm.

**Figure 4:**
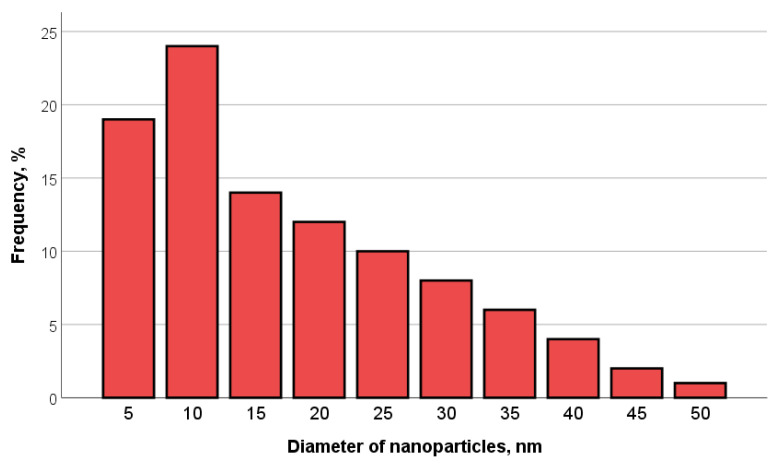
Particle size distribution for CeO_2_-II powder.

CeO_2_-II powder (Figure 3) also contained agglomerates, but the size of the agglomerates was two times smaller than that of the previous sample. The size of the main mass of the agglomerates was in the range of 50–800 nm. The shape of the agglomerates was loose. The particles composing the agglomerate were finely dispersed and crystalline. The agglomerates consisted of fairly homogeneous particles in size. The general appearance of the agglomerates is shown in Figure 3a. The electronogram obtained from this agglomerate, shown in Figure 3b, showed that the arrangement of the diffraction maxima on the ring electronogram corresponded to the CeO_2_ phase. The blurring of the rings in the electronogram also indicated the fine structure of the powder. Figure 3e–f shows a high-resolution electron microscopic image. Chaotically oriented nanocrystallites, mostly 5–10 nm in size, with a fairly perfect structure can be seen. Almost all the crystallites showed traces of atomic planes. Obtaining the same diffraction patterns from the different agglomerates indicated that the revealed patterns were common for different parts of the investigated sample, in which chaotically oriented nanocrystallites were observed. The main mass of the NPs (43%) was up to 10 nm in size, mostly 5–10 nm (Figure 4).

**Figure 5:**
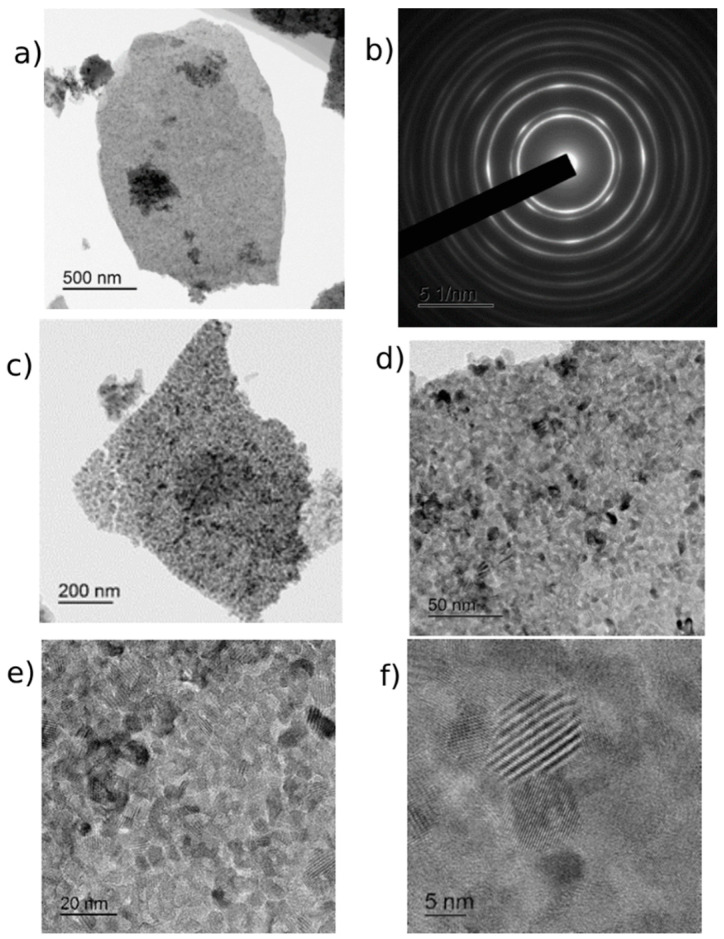
TEM image of CeO_2_-III powder particles: (**a**) overview image of agglomerate and (**b**) its electronogram; (**c**–**f**) enlarged image of individual grains with ruler of different sizes up to 5 nm.

**Figure 6:**
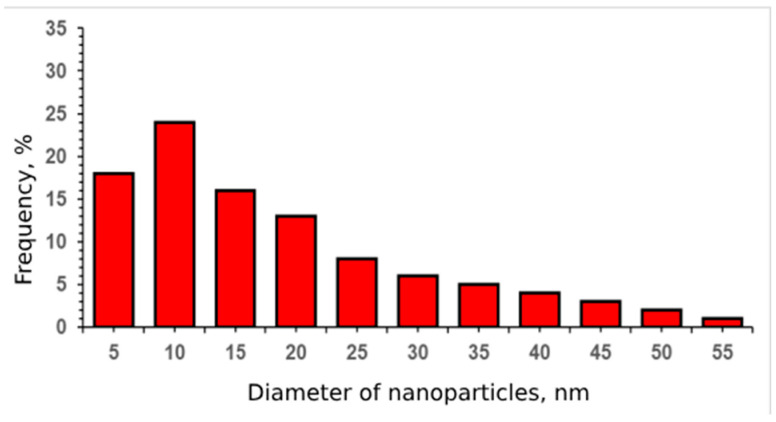
Grain size distribution for CeO_2_-III powder.

According to the TEM data, CeO_2_-III powder (Figure 5) represented agglomerates of different shapes and sizes from tens of nm to the micrometer. The agglomerates had complex shapes and consisted of fused, finely dispersed particles. A general view of the agglomerates is shown in Figure 5a, and Figure 5b shows the diffraction pattern obtained from an agglomerate. The location of the diffraction maxima on the ring electronogram corresponded to the CeO_2_ phase. The blurring of the rings on the electronogram indicated the finely dispersed structure of the powder. The study of the structure showed that the agglomerates consisted of chaotically oriented crystallites with an average size of 5–10 nm (as in CeO_2_-II powder). Practically in all the CeO_2_-III nanocrystallites, the traces of the atomic planes were resolved. The shape of the particles was mainly spherical. The crystallites had rather perfect structures. In Figure 5e–f, high-resolution images of CeO_2_ particles are shown, demonstrating that most of the nanoceria were 5–10 nm in diameter, which is confirmed in Figure 6.

#### 3.1.2. TEM of CeO_2_ Samples from Different Levels

**Figure 7:**
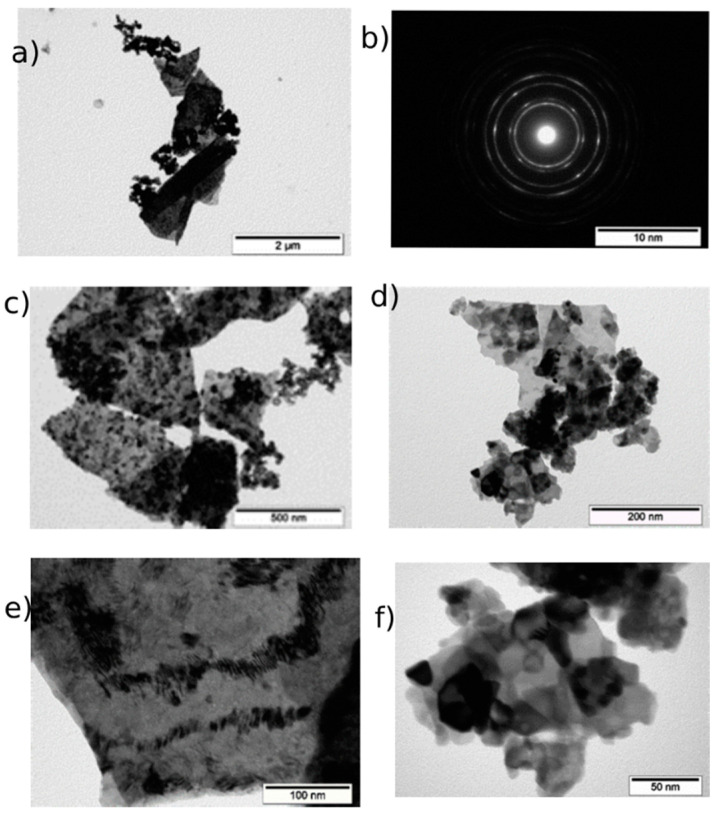
TEM images of CeO_2_-I sols in the lower fraction: (**a**) overview image of large aglomerate and (**b**) electronogram from this agglomerate; (**c**–**f**) enlarged image of individual grains with ruler of different sizes.

The study of CeO_2_-I sol after 72 h of sedimentation at the level of the lower fraction showed that the sample was highly crystalline and polycrystalline, consisting of polymorphic microparticles with the average size of several tens of nanometers. These crystallites (as part of microparticles) were of different morphologies, but most likely belonged to the same phase, which was confirmed by the electronograms. A large number of agglomerates, up to several microns in size, attracted attention. The microparticles were semi-dispersed with a size range from tens of nm to several microns. It was difficult to determine the exact linear dimensions of the NPs due to their polymorphic nature (Figure 7).

**Figure 8:**
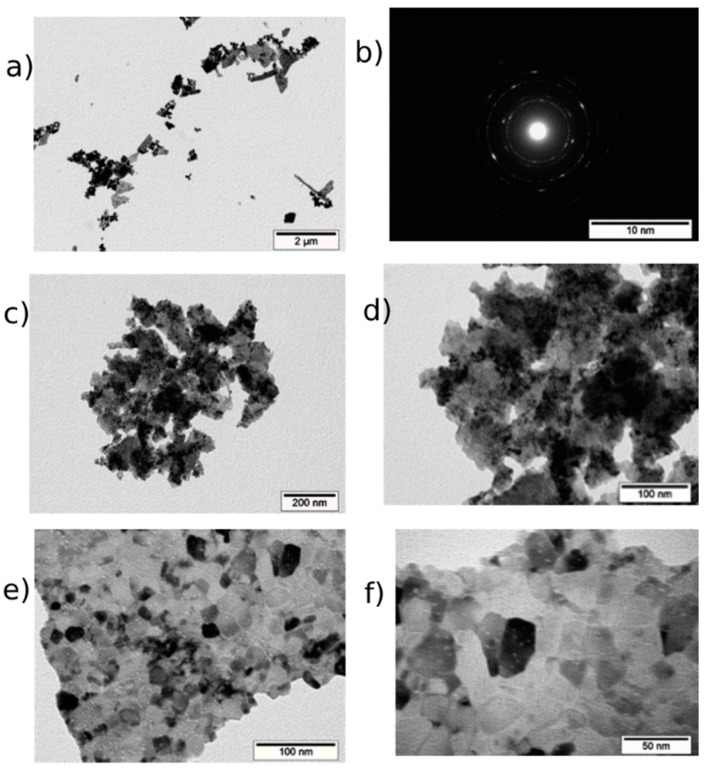
TEM images of a sample of CeO_2_-I sol from the middle. (**a**–**c**) Light-field (left), dark-field (middle) PEM images, and electronograms (right) of this agglomerate; (**d**–**f**) photographs at different magnifications.

**Figure 9:**
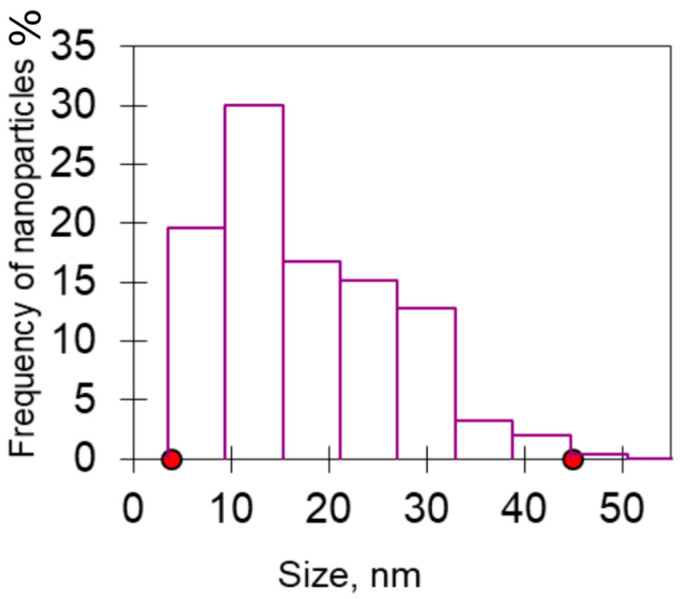
Histogram of crystallite size distribution of middle part of CeO_2_ sol.

The sample of the middle fraction (Figure 8) was highly polycrystalline (similar to the sample from the lower fraction) and consisted of microparticles, which, in turn, were composed of polymorphic NPs with sizes of several tens of nanometers. At the same time, the size of the agglomerates was significantly smaller than in the lower fraction. In addition to reducing the number of agglomerates, their size had also been reduced. In contrast to the lower fraction, no agglomerates larger than 800 nm were detected in the middle fraction. The visualization of the nanoceria at a high resolution showed that some crystallites contained mesopores, which might be, for example, a consequence of the dehydroxylation and compactization of the phase during synthesis. From the obtained micrographs it was evident that the sample had a polydisperse and polycrystalline structure with an average crystallite size of 16 ± 9 nm (Figure 9).

**Figure 10:**
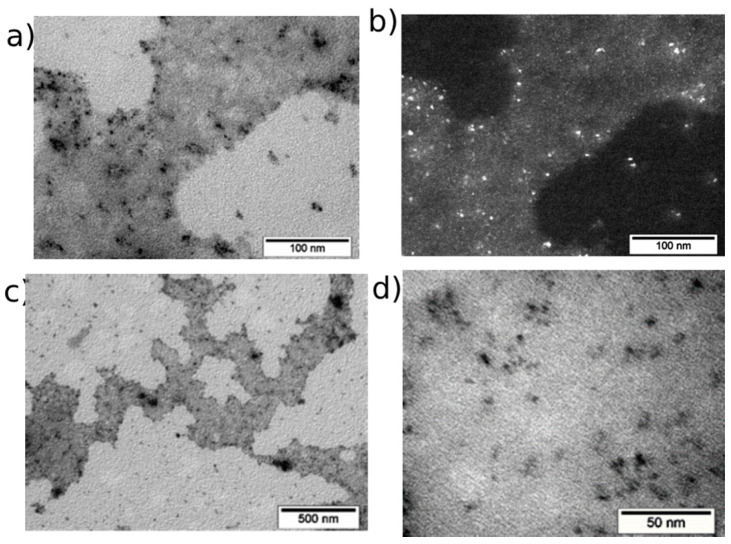
Brightfield (**a**,**c**,**d**) and darkfield (**b**) TEM images at different magnifications from the upper nano-ceria fraction (top 10% of sol after sedimentation).

In contrast to the lower and middle levels of the nanoceria sol, no agglomerates larger than 20 nm were detected in the upper fraction. The sample was mainly represented by individual scattered NPs. The size of the NPs was very small and did not exceed 5 nm, averaging 1–2 nm. The NPs were monodispersed and distributed in an amorphous matrix, which was found in all the grid cells in the study of this sample. There were also NPs that were outside this matrix, but the proportion of such nanoceria was much smaller (up to 20%) (Figure 10).

### 3.2. Determination of Cerium Dioxide Concentration at Different Levels of Sol After Sedimentation

Gravimetrically, it was found that the upper fraction of the CeO_2_-I sol (in the top 10% of the sol) contained nanoceria at a concentration of 0.12 g/L (7 × 10^−4^ M), i.e., 0.7% of the initial nanopowder. Since we were not satisfied with the practical yield of the upper fraction, we performed several additional studies to determine the optimal level of nanoceria sol intake for subsequent biological experiments.

The middle fraction investigated by the TEM contained 3.7 g/L nanoceria (2.1 × 10^−2^ M). The lower fraction was not investigated, as it was recognized as unsuitable for biomedical development. However, we were not satisfied with the results of the middle fraction on TEM, so the upper and middle fractions of the sol (smaller upper half, at the level of the upper 40–45% of the nanoceria sol fractions) were taken for further biological studies to develop regenerative preparation. The gravimetry results showed that the concentrations in this case were CeO_2_-I at 2.5 g/L, CeO_2_-II at 3.2 g/L, and CeO_2_-III at 3.1 g/L, which corresponded to 0.01–0.02 M (practical yield: 14–19% of the obtained nanoceria powder, according to the different modifications of nanoceria synthesis).

### 3.3. Results of X-Ray Diffraction Analysis

The upper fractions (up to 45%) of the sols were taken and dried for this study.

The results of the X-ray diffraction analysis indicated the presence of only one phase in all the samples—cerium (IV) oxide with a cubic face-centered crystal lattice (space group Fm-3 m). The interplanar distances were in good agreement with the families of planes from the standard CeO_2_ map. The size of the coherent scattering region (NPs size) was 12.4 ± 3.0 nm for CeO_2_-I; 9.1 ± 3.0 nm for CeO_2_-II; and 6.7 ± 1.4 nm for CeO_2_-III, respectively. The values of microdeformation (strain) were 0.11 ± 0.06% for CeO_2_-I, 0.55 ± 0.04% for CeO_2_-II, and 0.34 ± 0.05% for CeO_2_-III, which confirmed the polycrystalline nature of the samples and the multiple defects in the crystal structure.

**Figure 11:**
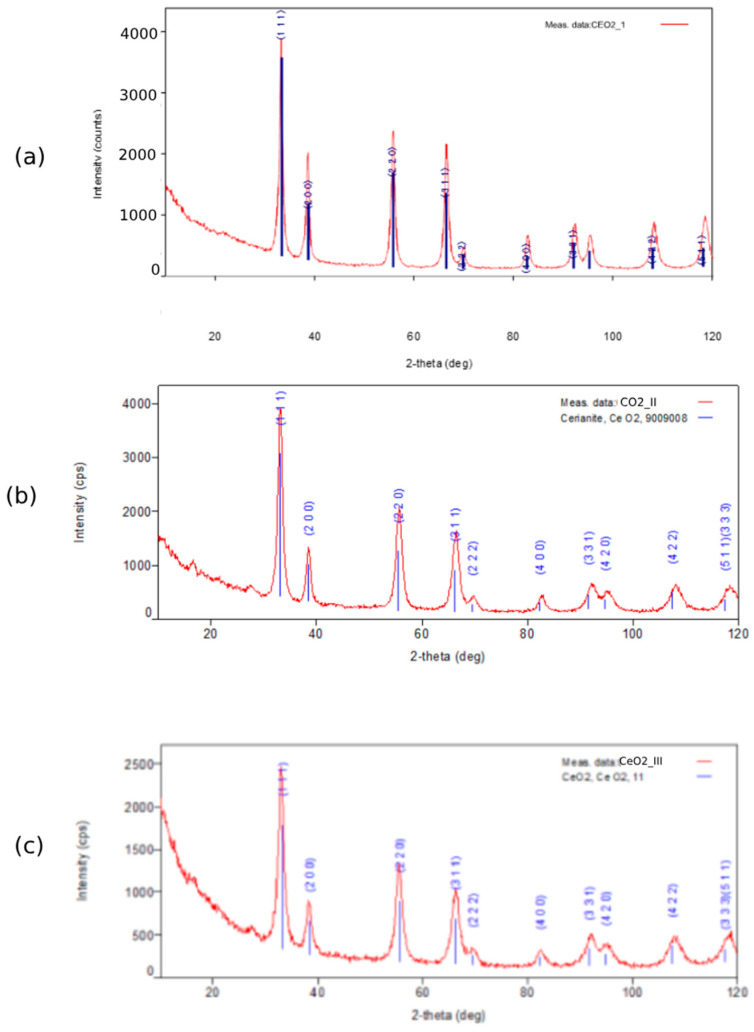
X-ray diffraction analysis patterns of CeO_2_-I (**a**), CeO_2_-II (**b**), CeO_2_-III (**c**).

Thus, in terms of NPs’ size indices, the advantage of CeO_2_-II and CeO_2_-III powders over CeO_2_-I was revealed (a size reduction of 1.36 times for CeO_2_-II and 1.85 times for CeO_2_-III, respectively, *p* < 0.05). In terms of the strain index, the most pronounced strains were found for the CeO_2_-II sample, which, on average, were 5 times larger than for CeO_2_-I (*p* < 0.05) and 1.62 times larger than for CeO_2_-III (Figure 11; Table 2).

### 3.4. Results of Raman Spectroscopy

**Figure 12:**
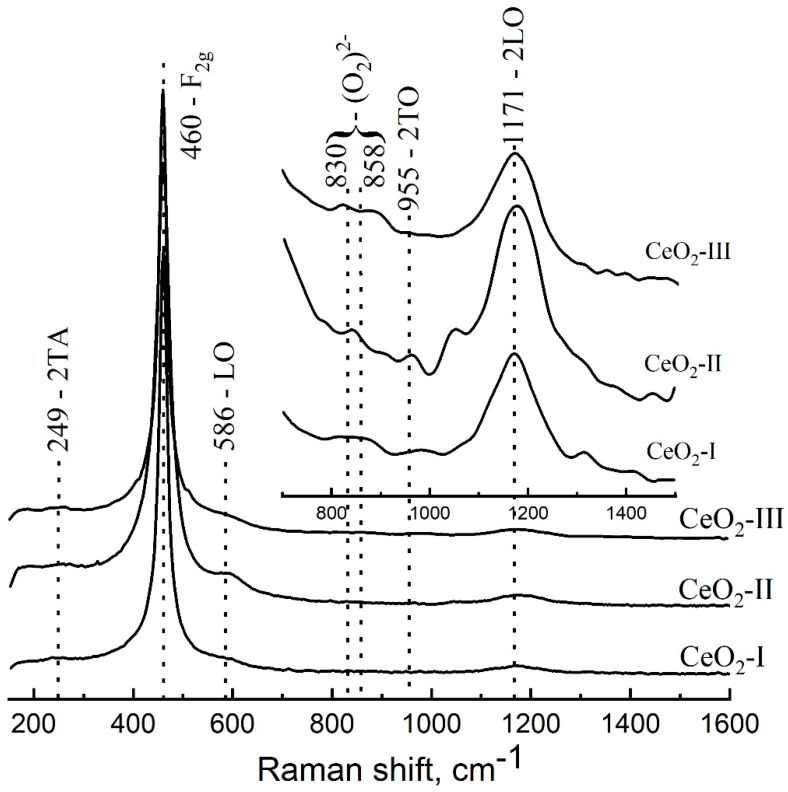
Raman spectra of CeO_2_ samples.

The samples of cerium oxide powders that deposited in a thick layer on the surface of a single-crystal silicon substrate were studied using Raman spectroscopy. The Raman scattering spectra of the CeO_2_-I, CeO_2_-II, and CeO_2_-III samples are shown in Figure 12.

The appearance of the spectrum obtained in terms of the fundamental and total vibrations of the crystal lattice is in good agreement with the spectra of both the bulk crystals and the CeO_2_ NPs [48,49,50]. Group–theoretical analysis for the cubic crystal lattice of the CeO_2_ of the fluorite type (s.g. 225, $Fm\bar{3}m$) shows that one of the most intense vibrations for such a structure is a triply degenerate Raman-active optical phonon with F_2g_ symmetry, which, at room temperature, is a manifestation of the stretching vibrations in the complex formed by the cerium atom at the cube vertex and eight oxygen atoms bound to it [48], and is experimentally observed near 460 cm^−1^ [48,49,50]. In addition, the measured spectrum of our sample contained a number of other normal and multiple modes: 2TA (249 cm^−1^), LO (586 cm^−1^), and 2LO (1171 cm^−1^). Vibrations are associated with the presence of structural defects of the crystal lattice, oxygen vacancies (broad shoulder in the region of 530–650 cm^−1^), and the Ce^3+^ defects associated with them. In addition, the spectra showed oscillations (830 and 858 cm^−1^) caused by the formation of peroxoforms on the particle surface as a result of oxygen adsorption on the surface defects [49]. The presence of the last two peaks indicated the existence of at least two types of defects on the particle surface that adsorbed oxygen with slightly different coupling force constants. The full width at half maximum (FWHM) of the F_2g_ mode can be used to estimate the average size of CeO_2_ NPs or crystallites. Thus, for commercial CeO_2_ samples, the dependence of the FWHM value on the average particle size was established in [50]: FWHM [cm^−1^] = 10.6 + 307.2/<D> [nm]. For our CeO_2_-II sample, FWHM = 32.5 cm^−1^, therefore <D> ≈ 14 nm, which was in good agreement with the results of the TEM studies. It is also worth noting that for the CeO_2_-II sample, the 2TO mode (about 955 cm^−1^) was clearly observed, which indicated a significant violation of the lattice symmetry due to the defectiveness of the structure, since the TO mode and its multiples are normally forbidden.

The full width at half maximum (FWHM) of the F_2g_ mode can be used to evaluate the degree of crystallinity of the CeO_2_ samples. Thus, for CeO_2_-II, the FWHM of the F_2g_ peak was the largest, 30 ± 1 cm^−1^, while for the CeO_2_-I and CeO_2_-III samples it was 20 ± 1 cm^−1^ and 18 ± 1 cm^−1^, respectively. The large FWHM for the CeO_2_-II indicated a high concentration of structural defects.

### 3.5. Mass Spectrometry Results

At the end of the physicochemical studies, mass spectrometry was performed. It was determined that the content of the main substance (CeO_2_) amounted to 99.99% in all powders, which indicated that a high-purity nanoceria powder was obtained (Table 3).

### 3.6. Results of Studies on Cell Lines

#### 3.6.1. Results of the Effect of CeO_2_ Nanoparticles Obtained Under Different Synthesis Conditions and in Different Concentrations on the Biocompatibility, Metabolic, and Proliferative Activity of Human Fibroblasts

**Figure 13:**
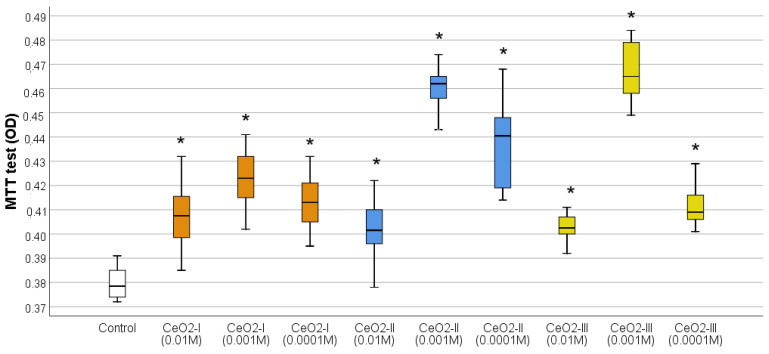
Effect of different methods and concentrations of cerium dioxide nanoparticles on metabolic activity of human fibroblasts in MTT test (ANOVA OD:F = 101.418; df 9, *p* < 0.001; *—different from control at *p* < 0.001).

The MTT test results showed that all the nanoceria samples at all the concentrations significantly stimulated fibroblast metabolism. The greatest difference from the control for all the variations of NP synthesis was registered at the concentration of 10^−3^ M (relative to the control, the OD indicator at co-cultivation with CeO_2_-I 10^−3^ M was, on average, 1.11 times higher, CeO_2_-II was 1.21 times higher, and CeO_2_-III was 1.23 times higher, *p* < 0.01) (Figure 13; Table 4).

According to the Duncan test, the best samples were CeO_2_-II and CeO_2_-III at concentrations of 10^−3^ M, which significantly stimulated fibroblast metabolism to a greater extent, significantly different from all the other subgroups (*p* < 0.01). Moreover, these samples of CeO_2_-II and CeO_2_-III at concentrations of 10^−3^ M were comparable to each other. The second place was CeO_2_-II at a concentration of 10^−4^ M, which was significantly different from the next level, 3, with the best stimulation of fibroblasts (CeO_2_-I at a concentration of 10^−3^ M).

**Figure 14:**
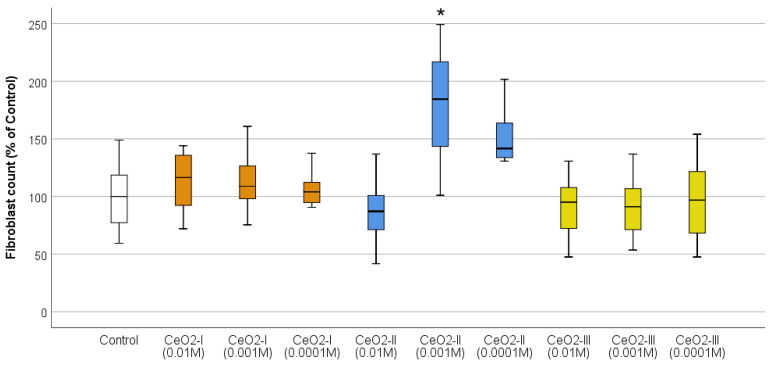
Effect of nanoceria production methodology and its concentrations on proliferative activity of fibroblasts (BJTERT cell line) by direct cell counting using automated cell counter. Mean percentages from control are presented (ANOVA OD:F = 5.216; df 9, *p* < 0.001; difference from control at *—*p* < 0.001; Bonferroni and Dunnett tests).

Cell counting after 72 h of co-cultivation established the clear advantage of CeO_2_-II at a concentration of 10^−3^ M, at which a stimulation of human fibroblast proliferation was registered by an average of 179 ± 52% relative to the control (*p* < 0.001). According to the ANOVA Bonferroni post hoc test, statistically significant differences in the number of fibroblasts CeO_2_-II at a concentration of 10^−3^ M were registered from all the other studied groups by an average of 66–92% (*p* < 0.05), except for the concentration of 10^−4^ M CeO_2_-II, at which the stimulation of cell division was registered by an average of 142 ± 46% relative to the control. In the remaining groups, no statistically significant differences were recorded in the cell number relative to control (Figure 14; Table 5), demonstrating the advantage of CeO_2_-II at a concentration of 10^−3^ M.

Thus, CeO_2_-II at a concentration of 10^−3^ M had the best effect on stimulating the metabolic and proliferative activity of human fibroblasts, which provided a rationale for selecting this particular variant of nanoceria for the further studies of it as a prototype wound-healing agent.

**Figure 15:**
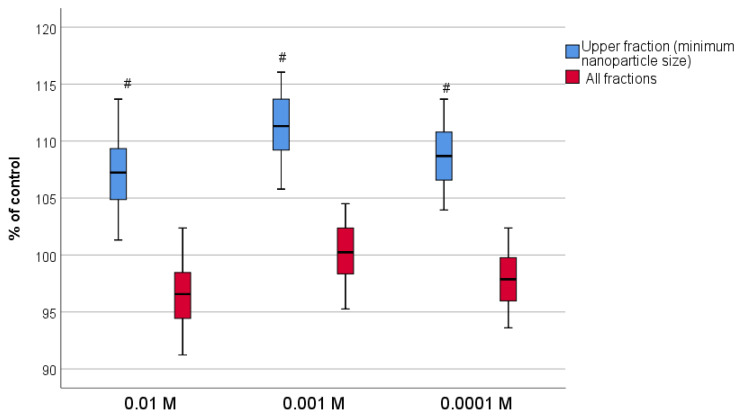
Comparison of MTT test results on fibroblasts during nanoceria synthesis with and without sedimentation (^#^—different between subgroups at *p* < 0.001).

**Figure 16:**
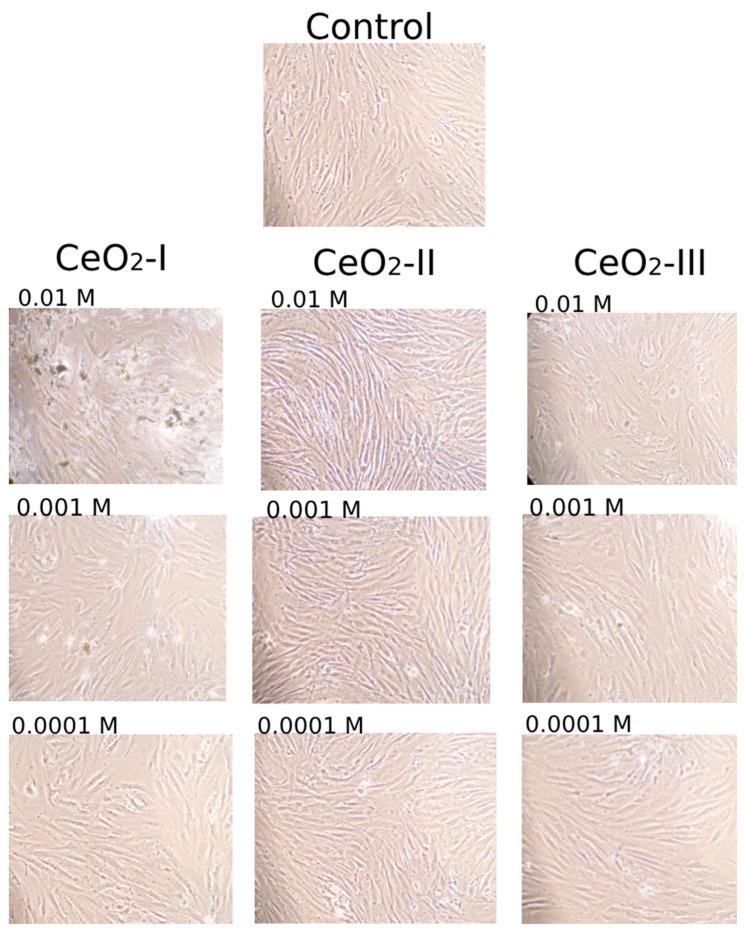
Photographs of fibroblasts after 72 h of co-cultivation with CeO_2_ of different synthesis modifications and at different concentrations (magnification ×20).

The MTT test results were evaluated at different levels of CeO_2_-I sol fractions, which were characterized by the largest size of the NPs. A significant difference from the norm was found in the OD index, which increased when co-cultivated with the smallest size NPs (the upper half of the fraction, up to 40–45% after sedimentation). When examining a sample of CeO_2_-I without the sedimentation step (i.e., a suspension containing all three fractions including large agglomerates), no statistically significant difference from the control was found at any of the tested concentrations 10^−2^–10^−4^ M (Figure 15). It was not possible to perform cell counting due to the large size of agglomerates at high concentrations of non-sedimented nanoceria, i.e., the sol without sufficient sedimentation did not allow the visualization of fibroblasts and quantitative counting (Figure 16).

The analysis of the percentage of dead cells when co-cultured with the upper half of the sedimented nanoceria showed no cytotoxicity. The percentage of the dead cells did not exceed 5% and was detected in up to one-third of the samples, which testified in favor of the biocompatibility of all the CeO_2_ samples studied by us, obtained under different modifications of synthesis (Figure 16).

#### 3.6.2. Results of Studies of the Effect of CeO_2_ Sols Obtained Under Different Synthesis Conditions and at Different Concentrations on Biocompatibility, Metabolic, and the Proliferative Activity of Human Keratinocytes

**Figure 17:**
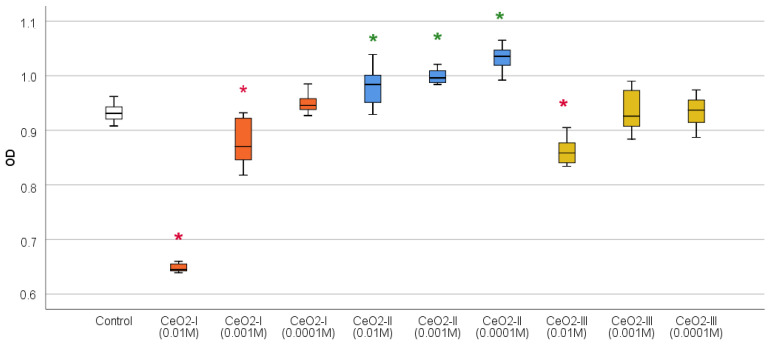
Effect of different methods and concentrations of cerium dioxide nanoparticles on metabolic activity of human keratinocytes in MTT test (ANOVA OD: F = 195.19; df 9, *p* < 0.001; * different from control at *p* < 0.001; red *—significant depression, green *—significant stimulation of cells relative to Control).

The results of an MTT test on human keratinocytes showed that high concentrations (10^−2^ M) of the samples CeO_2_-I and CeO_2_-III suppressed the metabolism and cellular activity of keratinocytes 1.44 times (*p* < 0.001) and 1.08 times (*p* < 0.05), respectively. This might be due to the negative effect of agglomerates, the frequency and size of which were maximal in the CeO_2_-I sample, at which the suppression of cellular activity by an average of 1.06 times (*p* < 0.05) was detected at a concentration of 10^−3^ M. Only when co-cultured with all the concentrations of CeO_2_-II (10^−2^ to 10^−3^ M) was a stimulation of keratinocyte metabolism recorded, and this stimulation was proportional to the degree of dilution. The OD index was significantly higher than in the control, on average, by 1.05 times at the concentration of CeO_2_-II 10^−2^ M, by 1.07 times at 10^−3^ M, and by 1.10 times at 10^−4^ M (*p* < 0.01). The MTT test values of the CeO_2_-II groups at concentrations of 10^−3^ M and 10^−4^ M were not statistically different from each other, with 10^−4^ M and 10^−3^ M CeO_2_-II being statistically significantly different from all the concentration subgroups of CeO_2_-I and CeO_2_-III (Bonferroni post hoc test, *p* < 0.01) (Figure 17; Table 6).

According to the Duncan test data, the best sample was the CeO_2_-II sample at a concentration of 10^−4^ M, and the concentrations of 10^−3^ M and 10^−2^ M CeO_2_-II were in second place.

**Figure 18:**
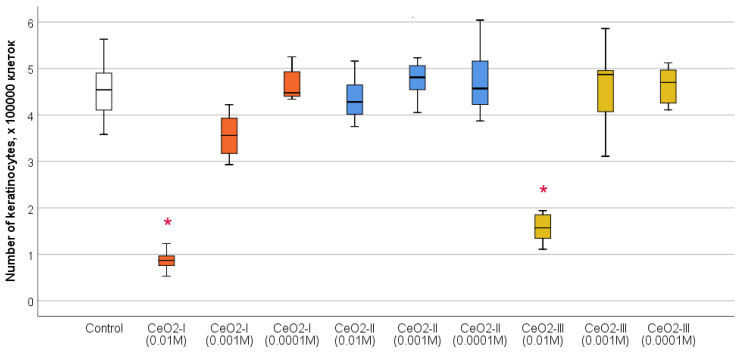
Effect of nanoceria synthesis methodology and its concentrations on proliferative activity of keratinocytes by direct cell counting using an automatic cell counter. Mean percentages from control are presented (ANOVA OD: F = 31.852; df 9, *p* < 0.001; difference from control at * ; *p* < 0.001; Bonferroni and Dunnett tests).

Counting the number of keratinocytes using a cell counter established that, at high concentrations, (10^−2^ M) CeO_2_-I samples contributed to the reduction in cell population growth by 5.2 times on average (*p* < 0.01), as well as when co-cultured with CeO^2^-III at a concentration of 10^−2^ M (regression of proliferation by 2.9 times, *p* < 0.01). All the investigated concentrations of CeO_2_-II sol did not inhibit keratinocyte proliferation and did not affect cell growth and multiplication, which indicates the highest level of safety in a wide range of CeO_2_-II concentrations (Figure 18; Table 7).

**Figure 19:**
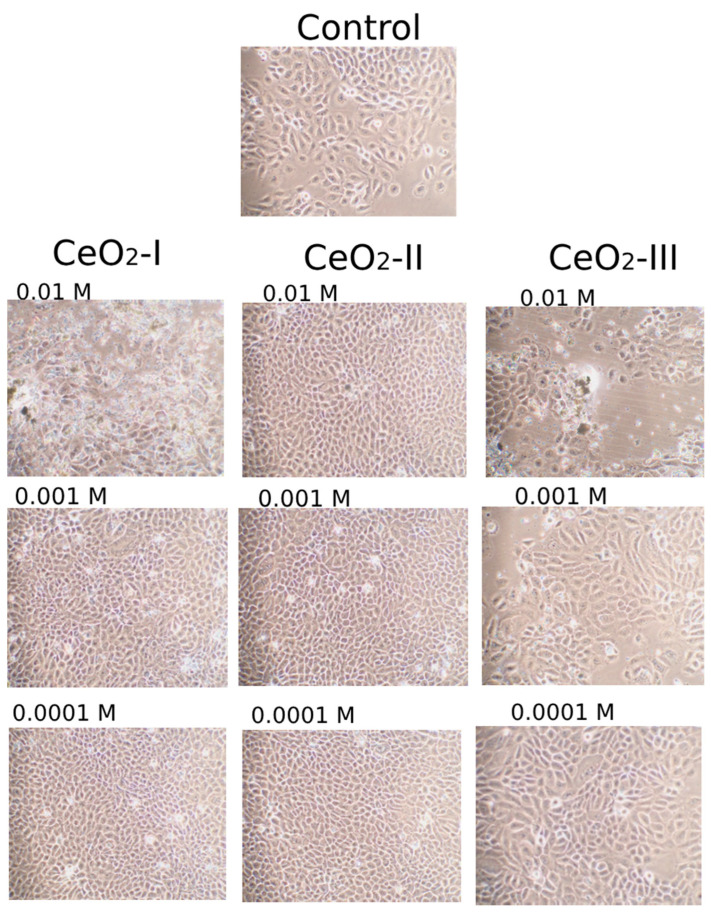
Photographs of keratinocytes after 72 h of co-cultivation with CeO_2_ of different synthesis modifications and at different concentrations. All the photographs were taken at the same magnification (×20). Visually, the seemingly smaller size of keratinocytes in the CeO_2_-II samples is associated with a higher concentration of more active keratinocytes, which are more pressed together compared to less active and rarer cells (due to this, they seem visually larger) in the CeO_2_-III sample.

In the assessment of cytotoxicity with the determination of live and dead cells, no statistically significant differences between the experimental groups were found. Dead cells were absent in most of the cases; they were found in single wells in minimal amounts (up to 10%). At the same time, it should be noted that dead cells were found more often in high concentrations (10^−2^ M) of CeO_2_-I and (less pronounced) CeO_2_-III. This was evidence in favor of the choice of a CeO_2_-II sample, which was characterized by a high level of biosafety, which was also confirmed by the visualization of the densest cell lining with the highest proliferation (Figure 19). In addition, we emphasized that the cerium dioxide NPs synthesized by all the methods we studied in concentrations from 10^−3^ M and below were biocompatible and non-toxic with respect to the different cell lines involved in the process of skin wound healing.

### 3.7. Results of Evaluation of Antimicrobial Activity Against Pseudomonas aeruginosa

**Figure 20:**
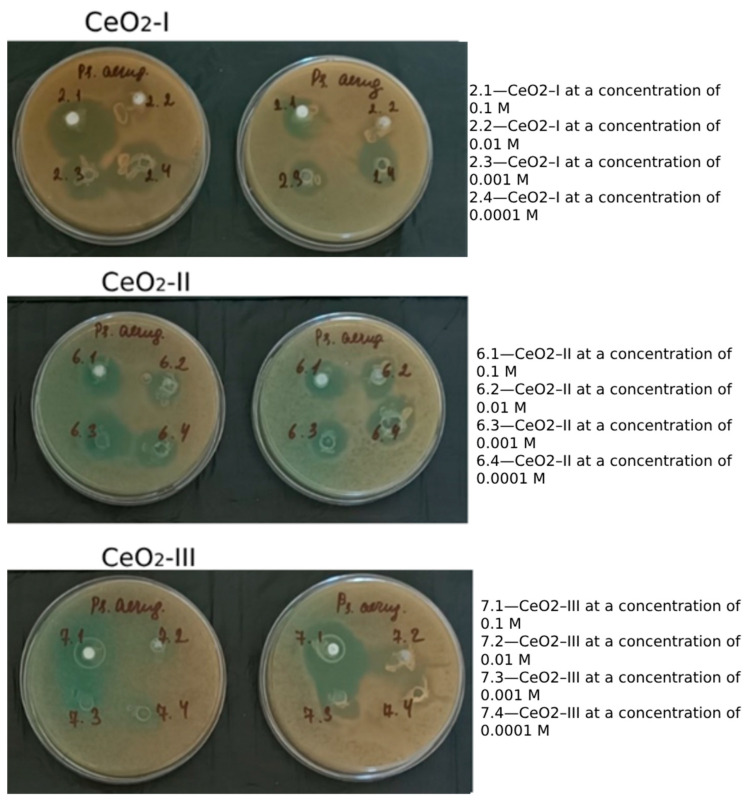
Growth retardation zones against *Pseudomonas aeruginosa* nanoceria obtained by different methods and at different concentrations.

It was found that all the samples of CeO_2_ in all the concentrations (10^−1^ M, 10^−2^ M, 10^−3^ M, 10^−4^ M) had antimicrobial activity against *Ps. aeruginosa* strain ATCC 9027. No significant differences were found in terms of the synthesis method or the concentration, varying on average from 14 mm to 23 mm (Figure 20).

## 4. Discussion

Cerium dioxide NPs are the most researched and widespread of all the rare-earth metal NPs, which are now widely used in various industrial applications. This is due, both to their unique physical and chemical properties, and to the fact that cerium is the most common and the cheapest of the rare-earth metals, with well-established production technology. All this contributed to the development of production technologies for the synthesis of CeO_2_ NPs, which in turn expands the areas and possibilities of their application in various fields of knowledge-intensive production.

The revealed regenerative, antimicrobial, and redox–active properties of CeO_2_ NPs [22,23,24,25,26,27,28,29,30,31,32,33,34,35,36,51,52,53,54,55,56,57,58,59,60,61,62,63] make them attractive as the basis for the creation of drugs. However, despite many years of research in this area, nanoceria has not yet been practically applied in biomedicine. This may be due to both the special requirements for the safety of new drugs and the ununified hundreds of modifications of the cerium dioxide synthesis techniques, which are not characterized by the accurate reproducibility and biological repeatability of their effects, which is unacceptable for drug creation.

In this regard, it is promising to select methodologies and technologies for the synthesis of nanoceria at factories that have many years of experience in their production on a production scale. One such factory that engaged in the synthesis and sale of rare-earth metal compounds, including CeO_2_ NPs, is LLC “LANHIT” (Moscow, Russia). The prospect of using nanoceria produced at LANHIT in large batches, not only for technical needs, but also for the biomedical sphere, is certainly attractive from a business point of view.

During the chemical part of our experiment of the synthesis of CeO_2_ NPs, we managed to determine the best parameters of synthesis methodology for obtaining particles of optimal sizes for the further creation of a wound-healing agent. The synthesis in each of the described groups was carried out three times, and in all cases, the physical and technical characteristics of the NPs did not differ between the groups. It was revealed that all the investigated CeO_2_ variants, obtained by different modifications of synthesis, possessed the same cubic crystal structure of fluorite; the sizes of the NPs and the agglomerates did not change at the repetition of the syntheses in different initial volumes and the purity of all the products was at the level of 99.99%. All this provides grounds to confirm the production reproducibility of a cheap non-toxic and simple technique for the synthesis of cerium dioxide NPs based on the thermal decomposition of cerium carbonate obtained by precipitation from nitrate aqueous solution. It should be noted that the synthesis of the starting substances (cerium nitrate and carbonate) was performed at the same certified production facility that has been using validated methods for more than 20 years, which can also be a guarantee for accurate reproducibility and for obtaining a chemically pure product.

The choice of nanoceria synthesis methodology was based, first, on the results of physicochemical and biological studies. The TEM carried out at the first stage showed that the best CeO_2_-II powder was obtained, the size of the main fraction of the NPs of which was 5–10 nm, and with the smallest number and size of agglomerates. CeO_2_-I powder represented large (up to several microns) agglomerates consisting of polycrystalline nanosized grains with an average diameter of 30 nm, which was three times larger than in other synthesis modifications. CeO_2_-III powder represented rather large agglomerates, but of smaller size than CeO_2_-I, consisting of NPs with an average grain size of 5–10 nm.

The largest size of the CeO_2_-I powder can be explained by the excessively high firing temperature (650 °C, which is 200 °C more than CeO_2_-II and CeO_2_-III) and its highest concentration of primary cerium nitrate. The larger agglomerates were related to the duration of calcination (3 h of CeO_2_-I and CeO_2_-III, which is 1.5 times longer than CeO_2_-II) as well as the temperature of the wash water, which was 2–3 times lower in the synthesis of CeO_2_-II compared to other methods used. Although the size of CeO_2_-II and CeO_2_-III were similar (5–10 nm on average) according to TEM data, and according to the XRD data for CeO_2_-II—9 nm on average—and CeO_2_-III—7 nm, CeO_2_-II was found to be the best in terms of biological activity. The high strain indicates the imperfect crystal structure of the CeO_2_-II sample, which in this case, is an advantage because it can lead to the higher reactivity of nanoceria. The antioxidant activity of cerium oxide is associated with the presence of structural defects on the surface of the particles, which leads to the appearance of reaction centers containing cerium ions capable of changing the degree of oxidation from 4+ to 3+ [13,53,55,63,64,65,66,67,68,69,70]. At the same time, the number of these centers correlates with the number of defects. The parameter of the number of microstresses directly indicates the specific number of defects in the crystal lattice and is the maximum for the CeO_2_–2 sample, which showed maximum biological activity.

Raman spectra reveal a large number of structural defects, such as oxygen vacancies and associated Ce^3+^. This is most typical for the CeO_2_-II sample. This is also confirmed by the parameter of the amount of microstrains in the crystal lattice.

In the cubic structure of CeO_2_ fluorite, due to the fact that oxygen ions are not densely packed, CeO_2_ can lose oxygen relatively easily, forming a large number of oxygen vacancies, while maintaining the basic structure of fluorite. Excess generated in this process is redistributed to the vacant cation levels, thereby changing its oxidation degree from Ce^4+^ to Ce^3+^. When charge centers are formed on the surface of CeO_2_, peroxoforms (reactive oxygen species) may well be adsorbed on them, and we observed these peaks on the Raman spectra (830 cm^−1^; 858 cm^−1^).

Structural defects in the surface layers of the crystal lattice, due to the implementation of the Ce^3+^ ↔ Ce^4+^ antioxidant cycle, were able to effectively neutralize active oxygen species (ROS), such as superoxide O_2_^−^, hydrogen peroxide H_2_O_2_, and a very dangerous short-lived active radical OH^*^. Thus, the higher the concentration of the structural defects on the CeO_2_ surface, the more effectively the particles were able to neutralize ROS. As experiments with cell cultures show, the CeO_2_-II sample exhibited maximum biological activity.

Consequently, different cerium dioxide NPs were synthesized from initially identical materials but under different conditions. The powders differed in their size and degree of dispersion of nanoparticles and their agglomerates. The highest dispersion of nanoparticle sizes was observed for the CeO_2_-I sample, while the lowest dispersion was observed for CeO_2_-II. The degree of agglomeration of the samples differed significantly; the CeO_2_-I and CeO_2_-III samples were more agglomerated, which was attributed to their different heat treatment conditions. Although the dispersion of the CeO_2_-II and CeO_2_-III NPs samples looked similar, the cell test results showed differences depending, not on the dispersion, i.e., particle size distribution, but on the size of the microstrains of the crystal lattice of the NPs, i.e., nanoceria redox activity.

Thus, higher temperatures of washing water (heating up to 30 °C and more) and drying, as well as increasing the temperature and time of calcination, promoted particle agglomeration. The obtained data are consistent with the results of other authors that show that the best CeO_2_ nanopowders are obtained at the lowest temperature effects [7,8,9,10,39,43,44,45], and the temperature regime affects all stages of synthesis. The higher the annealing temperature and duration were, the larger the particle size and the larger the size of the agglomerates.

Based on our data, we can conclude that when synthesizing CeO_2_ nanopowder in production conditions for biomedical purposes, it is advisable to use the calcination temperature of 450 °C for up to 2 h, and the temperature of washing water should not be higher than room temperature (20–25 °C).

As we have shown in earlier works [23,36,57], the biological effects of tissue regeneration, for which this study was conducted, are largely dependent on the size and concentration of CeO_2_ NPs. Therefore, despite the rapidity and simplicity of the technology of the selected synthesis of nanoceria, to obtain a quality biomedical product, we considered it necessary to carry out the second stage—the selection of the smallest NPs with the filtration of large agglomerates that can independently sediment under the action of gravity. For this purpose, we conducted a study of the aqueous suspension of nanoceria at different levels after 72 h sedimentation.

The results of the TEM of nanoceria suspension at different levels showed that in order to reduce the size of NPs in sols, it is advisable to perform the sedimentation of nanoceria for at least 72 h with a collection of the upper fractions (up to 10% of the volume from the top), where the NPs were dispersed without agglomerates and had the smallest size of 1–2 nm. However, the practical yield of such a fraction was extremely low (only 0.2%). In the study of the top 25% fraction, the concentration of nanoceria was 0.23 g/L, i.e., 1.3% of the initial concentration of nanopowder, which also did not satisfy us in terms of practical yield. From the point of view of economic efficiency for the production scale, it is expedient to bring the practical yield to the level of 15% of the initial mass of synthesized nanopowder; we searched for the optimal level of nanoceria selection in the aqueous suspension. The lower fraction contained a large number of agglomerates; the microparticles were polymorphic with a size range from tens of nm to several microns, so the lower fraction was definitely unsuitable for further biomedical research. In the middle fraction of the sol, the size of the NPs was, on average, two times smaller than in the unsedimented powder, but the size of the agglomerates did not satisfy us and limited the use of nanoceria suspension at the level of half of the fraction. Therefore, to perform subsequent biological studies for the development of a regenerative agent, we took the upper and middle fractions of the sols (smaller upper half, at the level of up to 40–45% of the volume from the top), where the concentration of CeO_2_ was 0.01–0.02 M (practical yield of 14.5–19.8% of the obtained nanoceria powder according to different synthesis modifications). The remaining nanomaterial (80–85% of nanoceria powder containing larger NPs and their agglomerates) could be realized for other types of industry, which would also increase the profitability.

Studies of the effects of nanoceria, when co-cultured with different cell lines, involved in the processes of skin wound regeneration, showed that nanoceria stimulated fibroblasts to a greater extent than keratinocytes. All the samples of nanoceria at all the concentrations stimulated fibroblast metabolism, but this was highest for CeO_2_-II and CeO_2_-III at concentrations of 10^−3^ M. At the same time, the numerical proliferation of human fibroblasts was significantly stimulated only by CeO_2_-II at a concentration of 10^−3^ M, which justified the choice of this particular subgroup (1 of 9) for the further development of regenerative nanomedicine.

Studies on human keratinocyte culture showed that only CeO_2_-II at all the concentrations we studied significantly stimulated cellular activity, increasing metabolism in the proportion to the dilution degree with the maximum level at a concentration of 10^−3^–10^−4^ M. Co-cultures with other variations of nanoceria had no stimulatory effect on the human keratinocytes. Keratinocyte proliferation was not enhanced when co-cultured with all the subgroups of nanoceria. On the contrary, high concentrations (10^−2^ M) of CeO_2_-I and CeO_2_-III might have cytotoxicity, which was expressed as the suppression of the metabolic and proliferative activity of keratinocytes.

At the same time, no reliable cell death was detected, even at high concentrations of nanoceria, which indicates the biocompatibility of all the tested samples. Dead cells were detected in single wells in minimal amounts (up to 10%). However, the tendency toward their increase in subgroups CeO_2_-I (10^−2^ M) and CeO_2_-III (10^−2^ M) does not allow us to recommend these samples for further biomedical application as a wound-healing agent.

Consequently, the greatest biological potential in relation to various human cell lines involved in the regeneration of skin wounds with the best biocompatibility is available in the technology of the synthesis of CeO_2_-II, with the best concentration range being of 0.001 M (up to 0.0001 M).

A microbiological study was conducted on the culture of *Pseudonos aeruginosa*. This microorganism has high resistance to all modern antibiotics and creates the greatest problems in clinical medicine. This study showed that all the modifications of CeO_2_ synthesis and in all concentrations (0.1 M–0.0001 M) had comparable antimicrobial activity against *Ps. aeruginosa*. The ability to suppress the growth and reproduction of one of the most problematic microorganisms, *Ps. aeruginosa*, is an additional argument for the use of synthesized nanoceria in surgical practice.

The mechanism of nanoceria’s antimicrobial action remains to be explored, as does why, at the same concentration, nanoceria is able to simultaneously inhibit Ps. aeruginosa reproduction and stimulate regeneration by enhancing cellular growth and activity. In our work, we only qualitatively determined the presence of antimicrobial activity. However, we did not conclude the dose dependence of nanoceria and its quantitative efficacy. An algorithm for selecting methods to study the antimicrobial properties of nanoceria, which is not capable of dissociation and thus has a very limited diffusion into agar, was presented earlier [60]. We plan to perform further studies using a gas chromatograph with mass spectrometry on a line of different microorganisms (quantitative microbial counting) with simultaneous studies to quantify the redox activity (pro- and anti-oxidation) of nanoceria. The screening method of diffusion into agar, which we used in this article, and which is generally accepted for standard pharmacopeia, showed the possibility of using nanoceria synthesized in production conditions in kilogram batches in the treatment of simulated wounds infected with *Ps. aeruginosa*. It is this animal study that we are now finalizing. However, these are topics for another article. Unfortunately, it is impossible to include everything in one publication. From our point of view, we have completely fulfilled the aims of this work.

Thus, it can be stated that it was possible to develop a method for the industrial synthesis of nanoceria, which can be used to produce drugs and medical devices containing nanoceria. CeO_2_-II at a final concentration of 10^−3^ M was chosen to create a prototype nanodrug of a wound-healing agent. It is interesting to note the fact that the same concentration of nanoceria 0.001 M (0.17 g/L by a mass of 900 °C of CeO_2_ burned) turned out to be the most optimal concentration, which was obtained in our work repeatedly, and with completely different synthesis methods, including the creation of nanoceria surrounded by a dextran coating or citrate coating [23,36].

## 5. Conclusions

We arrived at the following conclusions:

1. Changing any of the parameters in the process of nanoceria synthesis leads to a change in the physical and technical characteristics of the resulting nanomaterial, accompanied by a change in its biological activity. Increasing the calcination temperature of carbonate to 650 °C leads to an increase in the size of CeO_2_ NPs to an average of 30 nm, and decreasing the temperature to 450 °C leads to a decrease in the size of CeO_2_ NPs to an average of 5–10 nm. Decreasing the duration of calcination to 2 h is accompanied by a decrease in the agglomeration of NPs, and the agglomeration increases with increasing the duration of high temperature exposure;

2. When developing cerium dioxide nanopowder with the required technical characteristics on a production scale, it is advisable to apply the method of reducing the temperature at all the stages of nanoceria synthesis to the optimal level: to perform the calcination of carbonate at a temperature not exceeding 450 °C with a holding time of 2 h, for the temperature of washing water, use the temperature of 20 °C, which is optimal both in terms of technological simplicity and the quality of the resulting nanoceria for biomedical purposes;

3. To optimize the economic parameters of synthesis and obtain nanoparticles of optimal size, it is advisable to carry out at least 12 h sedimentation with sampling of up to 40–45% of the volume of the upper fraction of the initial 0.1 molar suspension;

4. The Raman spectroscopy spectra showed the presence of structural defects in the crystal lattice—oxygen vacancies and associated Ce^3+^ defects. The CeO_2_-II sample exhibited the largest full width at half maximum (FWHM) of the F2g mode, indicating a high concentration of structural defects. These defects may play a functional role in neutralizing active oxygen species on the particle surface;

5. The obtained nanoceria had antimicrobial activity against *Pseudomonas aeruginosa* at all the modifications of CeO_2_ synthesis and at all the concentrations (10^−1^ M–10^−4^ M);

6. Nanoceria stimulates fibroblasts to a greater extent than keratinocytes. All the variations of synthesized nanoceria at all the concentrations stimulated human fibroblast metabolism, but to the greatest extent, CeO_2_-II and CeO_2_-III at concentrations of 10^−3^ M, with only CeO_2_-II at a concentration of 10^−3^ M, significantly stimulated fibroblast proliferation after 72 h of co-culture. The metabolism of the human keratinocytes was stimulated only by CeO_2_-II at all the concentrations (10^−2^–10^−4^ M) in proportion to the degree of dilution with no significant effect on their proliferation;

7. The obtained effect of the stimulation of metabolism and the proliferation of cell cultures allows us to recommend CeO_2_-II at a concentration of 10^−3^ M to continue work on the development of a nanodrug that accelerates the regeneration of skin wounds. CeO_2_-II prepared by the method described above, at a concentration of 10^−3^ M (0.172 g/L~0.2 g/L), contains NPs with NP diameters up to 10 nm, which are non-toxic and have maximum biocompatibility and biological activity. It is this nanomaterial that will be used for toxicity studies and the evaluation of the effectiveness of wound treatment in animals.

## Figures and Tables

**Table 1:** Methods used for the synthesis of cerium dioxide nanoparticles under production conditions.

Indicator	CeO_2_-I	CeO_2_-II	CeO_2_-III
C[Ce(NO_3_)_3_], mol/L	0.3	0.145	0.058
C[Ce(NO_3_)_3_], g/L by content (CeO_2_)	50	25	10
t_water_; °C	40–50	20–25	30–40
t_drying_; °C	40	20	30
t_burning_; °C	650	450	450
T_exposition_; hours	3	2	3
Total volume	1 L	1 L	1 L
Volume Ce(NO_3_)_3_, mL /with concentration	200 mL /250 g/L (50 g/L in total volume)	200 mL /125 g/L (25 g/L in total volume)	200 mL /50 g/L (10 g/L in total volume)
Volume crystallization regulator NH_4_NO_3_ /with concentration	300 mL /375 g/L (112.5g/L in total volume)	550 mL /375 g/L (206.25 g/L in total volume)	700 mL /375 g/L (262.5 g/L in total volume)
Volume (NH_4_)_2_CO_3_ /with concentration	500 mL /200 g/L (i.e., 100 g/L)	250 mL /200 g/L (i.e., 50 g/L)	100 mL /200 g/L (i.e., 20 g/L)
Final yield (mass of nanoceria powder)	46.6 (93%)	22.4 (90%)	8.5 (85%)

**Table 2:** Results of X-ray diffraction analysis of nanoceria obtained by different synthesis modifications.

	CeO_2_-I	CeO_2_-II	CeO_2_-III
Formula/Space group	CeO_2_ 225:Fm-3m	CeO_2_ 225:Fm-3m	CeO_2_ 225:Fm-3m
Content (%)	100.0	100.0	100.0
Phase name (a × b × c)	5.4179(10)	5.4321(17)	5.4345(16)
alpha(deg), beta(deg), gamma(deg)	90.00	90.00	90.00
V(A^3)	159.03(5)	160.29(9)	160.50(8)
Crystallite size	12.4 ± 0.3	9.1 ± 0.3	6.68 ± 0.14
Strain (%)	0.11 ± 0.06	0.55 ± 0.04	0.34 ± 0.05

**Table 3:** Mass spectroscopy results, in parts per million (ppm).

Element	CeO_2_-I	CeO_2_-II	CeO_2_-III
Na	4	5	6
Mg	4	5	5
Al	10	10	10
Si	<10	<10	<10
R	<2	<2	<2
Cf	4	2	4
Sc	<1	<1	<1
Ni	<1	<1	<1
V	<1	<1	<1
Cr	<1	<1	<1
Mn	<1	<1	<1
Fe	5	2	3
Ni	<1	<1	<1
Cu	<1	<1	<1
Zn	<1	<1	<1
Y	<1	<1	<1
La	5	7	8
Pr	10	5	5
Nd	2	1	1
Sm	<0.1	<0.1	<0.1
Eu	<0.1	<0.1	<0.1
Gd	<0.1	<0.1	<0.1
Tb	<0.1	<0.1	<0.1
Dy	<0.1	<0.1	<0.1
Ho	<0.1	<0.1	<0.1
Er	<0.1	<0.1	<0.1
Tm	<0.1	<0.1	<0.1
Yb	<0.1	<0.1	<0.1
Lu	<0.1	<0.1	<0.1
Pb	<0.1	<0.1	<0.1
Bi	<0.1	<0.1	<0.1
Yh	<0.1	<0.1	<0.1
U	<0.1	<0.1	<0.1

**Table 4:** Descriptive statistics of MTT test results on human fibroblasts.

	Average	Standard Deviation	Standard Error	95% Confidence Interval for the Mean Value		Minimum	Maximum
				Lower Limit	Upper Limit		
Control	0.380	0.005	0.001	0.376	0.382	0.372	0.391
CeO_2_-I (0.01 M)	0.408	0.014	0.004	0.398	0.416	0.385	0.432
CeO_2_-I (0.001 M)	0.422	0.012	0.003	0.414	0.430	0.402	0.441
CeO_2_-I (0.0001 M)	0.413	0.011	0.003	0.406	0.419	0.395	0.432
CeO_2_-II (0.01 M)	0.401	0.012	0.003	0.394	0.407	0.378	0.422
CeO_2_-II (0.001 M)	0.460	0.010	0.002	0.455	0.464	0.442	0.474
CeO_2_-II (0.0001 M)	0.438	0.018	0.004	0.429	0.447	0.414	0.468
CeO_2_-III (0.01 M)	0.402	0.005	0.001	0.399	0.404	0.392	0.411
CeO_2_-III (0.001 M)	0.467	0.011	0.003	0.461	0.472	0.449	0.484
CeO_2_-III (0.0001 M)	0.412	0.009	0.002	0.407	0.417	0.401	0.438
Total	0.421	0.029	0.002	0.416	0.425	0.372	0.484

**Table 5:** Descriptive statistics of cell counting results on human fibroblasts.

	Average	Standard Deviation	Standard Error	95% Confidence Interval for the Mean Value		Minimum	Maxim
				Lower Limit	Upper Limit		
Control	100.0	31.39	11.86	70.95	129.01	59.37	148.94
CeO_2_-I (0.01 M)	113.23	25.20	7.28	97.22	129.25	72.00	144.00
CeO_2_-I (0.001 M)	112.381	24.99	7.21	96.50	128.26	75.43	160.86
CeO_2_-I (0.0001 M)	103.92	17.58	5.07	92.75	115.10	67.14	137.43
CeO_2_-II (0.01 M)	87.10	30.96	11.70	58.46	115.74	41.64	136.78
CeO_2_-II (0.001 M)	179.29	59.35	22.43	124.39	234.17	101.01	249.24
CeO_2_-II (0.0001 M)	141.64	46.38	17.53	98.74	184.53	53.50	201.62
CeO_2_-III (0.01 M)	90.46	30.77	11.62	62.01	118.92	47.52	130.70
CeO_2_-III (0.001 M)	91.07	29.83	11.27	63.48	118.65	53.50	136.78
CeO_2_-III (0.0001 M)	96.87	38.47	14.54	61.29	132.45	47.52	154.00
Total	111.29	40.05	4.34	102.65	119.92	41.64	249.24

**Table 6:** Descriptive statistics of MTT test results on human keratinocytes.

	Average	Standard Deviation	Standard Error	95% Confidence Interval for the Mean Value		Minimum	Maximum
				Lower Limit	Upper Limit		
Control	0.933	0.016	0.005	0.922	0.943	0.908	0.962
CeO_2_-I (0.01 M)	0.648	0.008	0.002	0.643	0.653	0.639	0.660
CeO_2_-I (0.001 M)	0.878	0.039	0.011	0.852	0.903	0.818	0.932
CeO_2_-I (0.0001 M)	0.949	0.018	0.005	0.938	0.961	0.927	0.985
CeO_2_-II (0.01 M)	0.982	0.037	0.011	0.958	1.005	0.929	1.039
CeO_2_-II (0.001 M)	0.998	0.013	0.004	0.990	1.007	0.984	1.021
CeO_2_-II (0.0001 M)	1.029	0.028	0.008	1.012	1.047	0.968	1.065
CeO_2_-III (0.01 M)	0.862	0.023	0.007	0.847	0.877	0.834	0.905
CeO_2_-III (0.001 M)	0.935	0.035	0.010	0.912	0.958	0.884	0.990
CeO_2_-III (0.0001 M)	0.935	0.026	0.008	0.918	0.952	0.887	0.974
Total	0.915	0.105	0.010	0.896	0.934	0.639	1.065

**Table 7:** Descriptive statistics of cell counting results on human keratinocytes.

	Average	Standard Deviation	Standard Error	95% Confidence Interval for the Mean Value		Minimum	Maximum
				Lower Limit	Upper Limit		
Control	4.54	0.68	0.26	3.90	5.17	3.58	5.63
CeO_2_-I (0.01 M)	0.87	0.22	0.08	0.66	1.07	0.53	1.23
CeO_2_-I (0.001 M)	3.56	0.49	0.19	3.10	4.01	2.93	4.22
CeO_2_-I (0.0001 M)	4.49	0.52	0.20	4.01	4.97	3.58	5.25
CeO_2_-II (0.01 M)	4.36	0.52	0.19	3.87	4.83	3.75	5.16
CeO_2_-II (0.001 M)	4.89	0.67	0.25	4.27	5.51	4.05	6.16
CeO_2_-II (0.0001 M)	4.75	0.79	0.30	4.01	5.49	3.87	6.04
CeO_2_-III (0.01 M)	1.57	0.33	0.13	1.26	1.88	1.11	1.94
CeO_2_-III (0.001 M)	4.55	0.93	0.35	3.70	5.41	3.11	5.86
CeO_2_-III (0.0001 M)	4.69	1.07	0.40	3.71	5.68	3.11	6.62
Total	3.8280	1.50	0.18	3.47	4.19	0.53	6.62

## Data Availability

Details regarding the data supporting the reported results can be found from the first authors.
